# Trends in Dietary Nutrients by Demographic Characteristics and BMI among US Adults, 2003–2016

**DOI:** 10.3390/nu11112617

**Published:** 2019-11-01

**Authors:** Shan Han, Lanlan Wu, Wenjie Wang, Na Li, Xiaoyan Wu

**Affiliations:** Department of Nutrition and Food Hygiene, the National Key Discipline, School of Public Health, Harbin Medical University, Harbin 150081, China; hss1128@126.com (S.H.); Wulanlan2017@126.com (L.W.); wangwenjie0522@126.com (W.W.); nana19860716@126.com (N.L.)

**Keywords:** dietary nutrients, temporal trend, US adults, dietary reference intakes, the Dietary Guidelines for Americans

## Abstract

Background: Limited data were available on trends of US dietary nutrients especially for specific subgroups; Methods: Dietary intakes of energy and 36 kinds of nutrients were analyzed in the National Health and Nutrition Examination Survey (NHANES) from 2003 to 2016 and by age and sex, socioeconomic status, race/ethnicity, and body mass index, which were evaluated by whether not they meet the dietary reference intakes (DRIs); Results: Significantly decreased trends were observed for carbohydrate, total sugars, fiber, calcium, magnesium, phosphorus, selenium, vitamin B_6_, E, K, and choline, while increased trends were observed for saturated fatty acids, iron, zinc, copper, potassium, sodium, vitamin B_1_, B_2_, B_12_, C and folate DFE (as dietary folate equivalents). A decreased trend of exceeding the estimated energy requirement was found. Population with low socioeconomic status and non-Hispanic blacks accounted for the largest proportion not meeting DRIs for most of nutrients; Conclusions: Most dietary nutrients were improved among US adults from 2003 to 2016 but were still far from optimal levels. Populations with low socioeconomic status and non-Hispanic blacks should be paid more attention to improve their dietary nutrient intake.

## 1. Introduction

Diet is one of the leading causes of deaths and disability-adjusted life-years [1]. In the United States, dietary risks accounted for more than 650,000 deaths per year and more than 5% of risk-attributable of cardiovascular diseases (CVD), neoplasms, diabetes, diet-related cancers, obesity, and of urogenital, blood, and endocrine diseases [2]. Understanding trends in dietary nutrient intake is crucial in informing national health priorities to improve diets and reduce the risk of diet-related diseases [3].

Although trends of US dietary nutrient intake have been investigated in previous studies, most of them have focused on changes in only one or some nutrients among restricted age or gender groups [4,5,6,7]. Comparisons of energy, calcium, iron, magnesium, zinc, folacin, vitamin B_6_ and E intakes were reported between 1977 and 1985 surveys of women in the US Department of Agriculture’s Continuing Survey of Food Intakes [8]. In addition to the above nutrients, macro-nutrients have also been reported among adults between the 1987 and 1992 National Health Interview Surveys (NHIS) [9], and a comparison of fat intake was reported between the National Health and Nutrition Examination Survey (NHANES) II (1980) and the NHANES (1988–1991) [10]. However, all these studies are focused on data collected before 2010. Specifically, the US Department of Agriculture (USDA) data reported in 2016 showed that 54% of Americans paid more attention to healthy eating compared with 20 years ago, but not fully embracing what they learn [11]. Until now, limited evidence has been available on the trends of dietary nutrient intake in US nationally representative samples collected across recent years, especially on how such trends varying according to specific subgroups, which can provide much information on informing corresponding interventions.

To comprehensively depict trends of US dietary nutrient intake across the last 14 years, we used data from seven surveys of the NHANES (2003–2004, 2005–2006, 2007–2008, 2009–2010, 2011–2012, 2013–2014, and 2015–2016) to examine temporal trends in total energy and 36 kinds of dietary nutrients consumed by US adults overall and by age and sex, socioeconomic status, body mass index (BMI), and race/ethnicity. Specifically, the dietary reference intakes (DRIs) were used to examine whether the specific nutrient intake met the recommended amount [12].

## 2. Materials and Methods 

### 2.1. Population

The NHANES is a series of nationally representative cross-sectional health examination surveys conducted by the National Center for Health Statistics of the Centers for Disease Control and Prevention (CDC). The necessary ethical approvals for the NHANES have been obtained by the National Center for Health Statistics and the data are publicly available via the National Center for Health Statistics (NCHS) website. Following an in-home interview, participants are scheduled to visit a mobile examination center (MEC) to complete a physical examination and a 24-h dietary recall, and to obtain blood samples for laboratory measurement. The present data analyses were based on two non-consecutive days through 24-h dietary recall interviews from seven cycles (2003–2004 through 2015–2016). Participants were excluded if they reported consuming 0 kcal/day or were pregnant or younger than 18 years old. At last, a total of 34,099 respondents were analyzed in the present study.

### 2.2. Dietary Intake Assessment and DRIs

Dietary nutrients and energy intake were determined from the USDA Survey Nutrient Database (12). Except for total energy intake, a total of 37 kinds of dietary nutrients, including protein (g/day), carbohydrates (g/day), total sugars (g/day), dietary fiber (g/day), total fat (g/day), saturated fatty acids (g/day), monounsaturated fatty acids (g/day), polyunsaturated fatty acids (g/day), cholesterol (mg/day), calcium (mg/day), phosphorus(mg/day), magnesium (mg/day), iron (mg/day), zinc (mg/day), copper (mg/day), sodium (mg/day), potassium (mg/day), selenium (µg/day), vitamin A (µg/day), vitamin B_1_ (mg/day), vitamin B_2_ (mg/day), vitamin B_3_ (mg/day), vitamin B_6_ (mg/day), vitamin B_12_ (µg/day), folate DFE (as dietary folate equivalents) (µg/day), vitamin C (mg/day), vitamin D (µg/day), vitamin E (mg/day), vitamin K (µg/day), choline (mg/day), alpha-carotene (µg/day), beta-carotene (µg/day), beta-cryptoxanthin (µg/day), lycopene (µg/day), lutein + zeaxanthin (µg/day), caffeine (mg/day), and theobromine (mg/day). 

In addition, the estimated energy requirement (EER) was calculated to assess whether participants consumed energy which might be larger than their basic requirements [13]. The physical activity level (PAL) was set at 1, 1.12, 1.27, 1.54 for men and 1, 1.14, 1.27, 1.45 for women in the present study, respectively (13). The EER was calculated as

EER = 864 − 9.72 × age (years) + physical activity level (PAL) × (14.2 × weight (kg) + 503 × height (meters)) (for men)(1)

EER = 387 − 7.31 × age (years) + PAL × (10.9 × weight (kg) + 660.7 × height (meters)) (for women)(2)

The values of DRIs for each specific nutrient were used in the present study to evaluate intake levels, including estimated average requirement (EAR), recommended dietary allowance (RDA), adequate intake (AI), tolerable upper intake level (UL), acceptable macronutrient distribution range (AMDR), and dietary guidelines recommended limit (DGA) [14].

### 2.3. Subgroup Assessment

Socioeconomic status were defined based on educational attainment (EA) and a poverty income ratio (PIR) and, thus, participants were classified into high (more than 12 completed years of EA and a PIR of at least 3.5), low (less than 12 years of EA and a PIR less than 1.30), and medium (others). BMI (kg/m^2^) was defined as weight divided by height squared and participants were classified into underweight (<18.5 kg/m^2^), normal (18.5–24.9 kg/m^2^), overweight (25.0–29.9 kg/m^2^), and obese (≥30 kg/m^2^).

Subgroup assessment included age and sex (men aged 19–45 years, men aged ≥ 46 years, women aged 19–45 years, and women aged ≥ 46 years), socioeconomic status (high, medium, and low), race/ethnicity (Hispanic, non-Hispanic white, non-Hispanic black, and other race), and BMI (underweight, normal weight, overweight, and obese). 

### 2.4. Statistical Analyses

The percentages being less than EAR, RDA, and AI, more than UL and DGA, or not within AMDR range were calculated for specific nutrients. The intakes of energy and each nutrient were presented as weighted mean (standard error). Survey-weighted linear regression was used to test for a linear trend with each dietary nutrient intake as independent variables and treating the survey year as a dependent variable. To assess the statistical heterogeneity of subgroup trends, survey weighted tests were used to test the trend relationship between years and categorical variables (age and gender, socioeconomic status, BMI, and race/ethnicity). Sensitivity analyses were conducted among participants (*n* = 32,635) who did not intake implausible energy intake: <500/>3500 kcal/day for women and <800/>4000 kcal/day for men.

All statistical analyses were performed with SPSS version 20 (SPSS Inc., Chicago, IL, US). Graphic production was carried out by adopting R version 3.0.3 (The R Foundation for Statistical Computing, Vienna, Austria). Two-sided *p* < 0.05 was considered statistically significant.

## 3. Results

The demographic characteristics of the respondents were shown in Table 1 and Figure S1.

### 3.1. Trends of Macro-Nutrients Not Meeting DRIs

Trends of calories from protein, carbohydrate, and lipid were not changed from 2003 to 2016 and the averages of them were in the range of AMDR being 17.9%, 54.7%, and 38.7%, respectively, while the trend of carbohydrate intake was significantly decreased from 265.14 mg/day to 241.08 mg/day (*p*_for linear trend_ < 0.001, Table 2).

**Figure 1:**
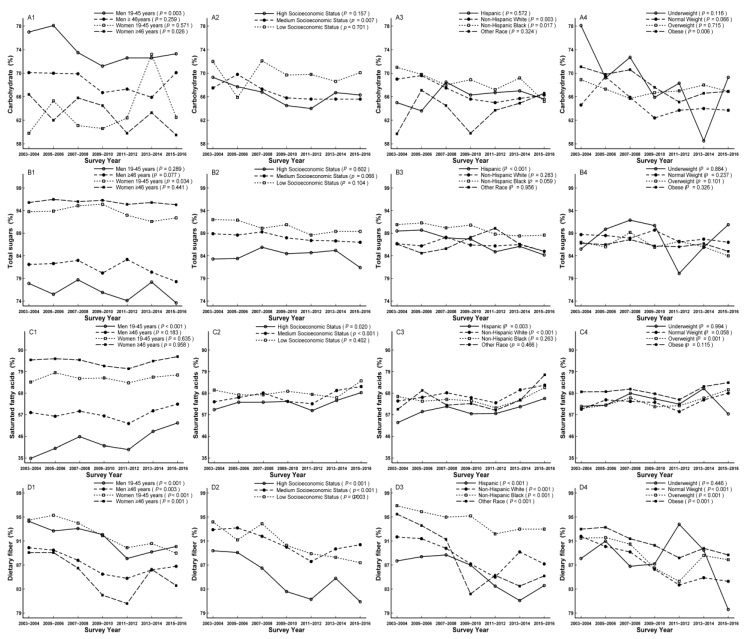
Trends of dietary carbohydrate (**A1**–**A4**), total sugars (**B1**–**B4**), saturated fatty acids (**C1**–**C4**), and fiber (**D1**–**D4**) by sex age, socioeconomic status, race/ethnicity, and body mass index (BMI) for percentage not meeting dietary reference intakes among US adults across seven surveys in the National Health and Nutrition Examination Survey (NHANES 2003–2016).

Trends of carbohydrate, protein, and fat were estimated by the percentage of population not meeting AMDR, with a decreased trend for carbohydrates from 68.4% to 66.1% (*p*_for linear trend_ = 0.001), but stable trends for protein and fat (*p*_for linear trend_ = 0.219 and 0.128, respectively) (Table 3). Although no modifications were found for protein and fat, the decreased trends were apparent in men aged 19–45 years for protein (*p*_for linear trend_ = 0.039) and high socioeconomic status for protein and total fat (*P*_for linear trend_ = 0.012 and 0.011, respectively; Table S1). The decreased trends of carbohydrate were apparent for men aged 19–45 years, women aged ≥ 46 years, participants with medium socioeconomic status, non-Hispanic whites, non-Hispanic blacks, and the obese (*p*_for linear trend_ = 0.003, 0.026, 0.007, 0.003, 0.017, and 0.006, respectively; Table S1). Men aged 19–45 years accounted for the largest proportion not meeting AMDR compared to their comparts (Figure 1A1–A4, Table S1).

A decreased trend for total sugars estimated by not meeting DGA was observed from 87.5% to 85.3% (*p*_for linear trend_ = 0.047, Table 3), which was also observed in its intake from 122.59 mg/day to 102.94 mg/day (*p*_for linear trend_ < 0.001, Table 2). This trend was apparent for women aged 19–45 years and Hispanics (*p*_for linear trend_ = 0.034 and < 0.001, respectively). Women aged ≥ 46 years and participants with low socioeconomic status accounted for the largest proportion not meeting DGA compared to their comparts (Figure 1B1–B4, Table S1). A decreased trend for dietary fiber estimated by not meeting AI was found from 92.0% to 87.1% (*p*_for linear trend_ < 0.001, Table 3), while an increased trend was observed in its intake from 15.67 mg/day to 17.33 mg/day (*p*_for linear trend_ < 0.001, Table 2). A decreased trend was observed in all subgroups except for underweight (*p*_for linear trend_ = 0.446). Women aged 19–45 years and non-Hispanic blacks accounted for the largest proportion not meeting AI compared to their comparts, while high socioeconomic status accounted for the lowest proportion (Figure 1D1–D4, Table S1).

An increased trend for saturated fatty acids estimated by not meeting DGA was observed from 62.8% to 70.6% (*p*_for linear trend_ < 0.001, Table 3). This trend was apparent for men aged 19–45 years, participants with high and medium socioeconomic status, Hispanics, non-Hispanic whites, and the overweight (*p*_for linear trend_ < 0.001, = 0.020, < 0.001, = 0.003, < 0.001, and = 0.001, respectively), and women aged ≥ 46 years accounted for the largest proportion not meeting DGA compared to their comparts (Figure 1C1–C4, Table S1). In addition, the increased trend was observed for polyunsaturated fatty acids intake from 17.47 to 19.04 mg/d (*p*_for linear trend_ < 0.001), while a decreased trend was found for monounsaturated fatty acids intake from 31.10 mg/d to 29.12 mg/d (*p*_for linear trend_ < 0.001) (Table 2).

### 3.2. Trends of Minerals Not Meeting DRIs

Trends of 7 kinds of minerals were estimated by not meeting RDA, with decreased trends for calcium, magnesium, phosphorus, and selenium, being from 70.2% to 64.5%, 81.1% to 74.4%, 9.8% to 8.6%, and 9.8% to 8.2%, (*P*_for linear trend_ < 0.001, < 0.001, = 0.011, and < 0.001, respectively), while increased trends for iron, zinc, and copper, being from 31.2% to 34.5%, 35.6% to 41.5%, and 28.8% to 30.7%, (*p*_for linear trend_ = 0.006, < 0.001, and < 0.001, respectively; Table 3).

The increased trends were observed for intakes of magnesium and selenium, being from 279.88 to 345.25 mg/day and 111.24 to 115.34 mg/day, (*P*_for linear trend_ < 0.001 and < 0.001, respectively), while decreased trends were observed for intakes of iron and zinc, being from 16.07 to 14.03 mg/day and 12.24 to 11.20 mg/day, (*p*_for linear trend_ < 0.001 and < 0.001, respectively). On the other hand, the increased trends were observed in intakes of calcium and phosphorus from 2003 to 2010 (880.28 to 1011.61 mg/day and 1335.34 to 1412.72 mg/day, respectively), but decreased slightly thereafter (Table 2).

**Figure 2:**
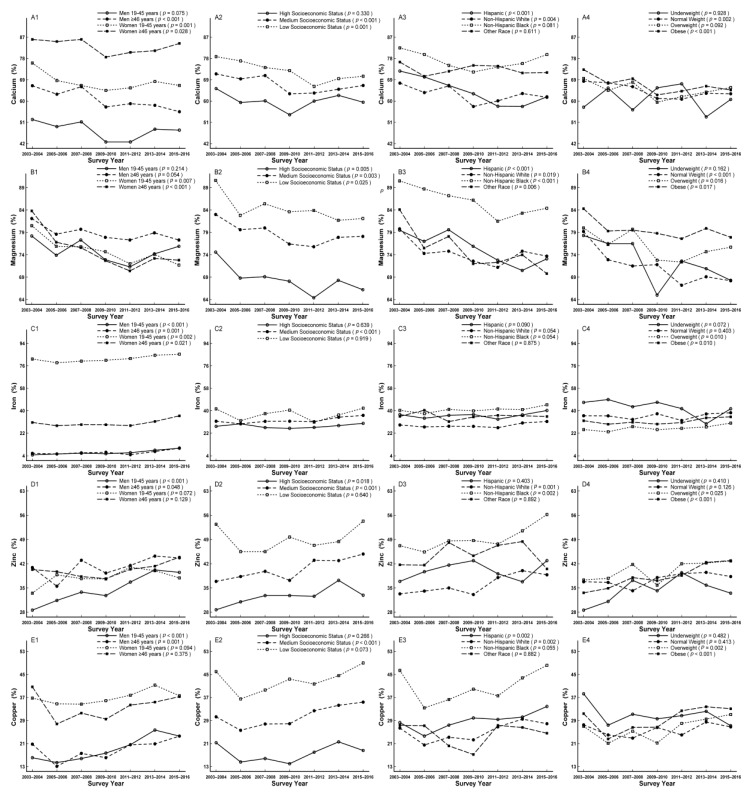
Trends of dietary calcium (**A1**–**A4**), magnesium (**B1**–**B4**), iron (**C1**–**C4**), zinc (**D1**–**D4**), and copper (**E1**–**E4**) by sex age, socioeconomic status, race/ethnicity, and body mass index (BMI) for percentage not meeting dietary reference intakes among US adults across seven surveys in the National Health and Nutrition Examination Survey (NHANES 2003–2016).

**Figure 3:**
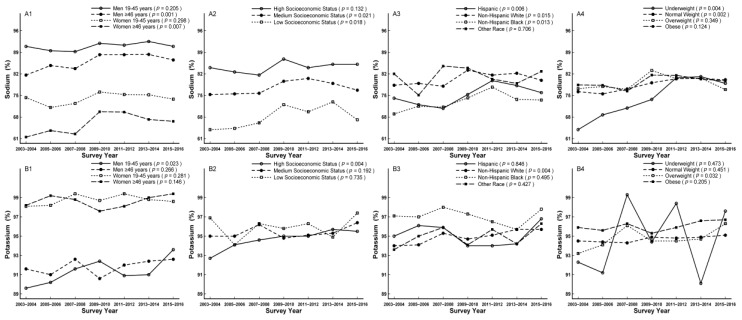
Trends of dietary sodium(**A1**–**A4**) and potassium (**B1**–**B4**) by sex, age, socioeconomic status, race/ethnicity, and body mass index (BMI) for percentage not meeting dietary reference intakes among US adults across seven surveys in the National Health and Nutrition Examination Survey (NHANES 2003–2016).

In subgroup analysis, the decreased trends were not observed in men aged 19–45 years, participants with high socioeconomic status, non-Hispanic blacks, other race, underweight, and overweight for calcium (*p*_for linear trend_ = 0.075, 0.330, 0.081, 0.611, 0.928, and 0.092, respectively), which were additionally not observed in men and underweight for magnesium (*p*_for linear trend_ = 0.214, 0.054 and 0.162, respectively) (Figure 2A1–A4,B1–B4). The increased trends were apparent in all sex and age groups, participants with medium socioeconomic status, overweight, and obese for iron (*p*_for linear trend_ < 0.001, = 0.001, 0.002, 0.021, < 0.001, = 0.010, and 0.010, respectively), in men, participants with high and medium socioeconomic status, non-Hispanic whites, non-Hispanic blacks, overweight, and obese for zinc (*p*_for linear trend_ < 0.001, = 0.048, = 0.018, < 0.001, = 0.001, 0.002, 0.025, and < 0.001, respectively), and in men, participants with medium socioeconomic status, Hispanics, non-Hispanic whites, overweight, and obese for copper (*p*_for linear trend_ < 0.001, = 0.001, < 0.001, = 0.002, 0.002, 0.002, and < 0.001, respectively) (Figure 2C1–C4,D1–D4,E1–E4). In addition, participants with low socioeconomic status and non-Hispanic blacks accounted for the largest proportion of not meeting RDA for calcium, magnesium, iron, zinc, and copper compared to their comparts, respectively (Figure 2A2,B2,C2,D2,E2; Table S1). Furthermore, women aged ≥ 46 years, men aged ≥ 46 years, and women aged 19–45 years accounted for the largest proportion of not meeting RDA for calcium, magnesium, and iron compared to their comparts, respectively (Figure 2A1,B1,C1; Table S1).The trend of potassium was estimated by not meeting AI with increased trend from 94.4% to 96.1% (*p*_for linear trend_ = 0.006, Table 3), which was apparent in men aged 19–45 years, participants with high socioeconomic status, non-Hispanic whites, and overweight (*p*_for linear trend_ = 0.023, 0.004, 0.004, and 0.032, respectively) (Figure 3B1–B4). In addition, women and non-Hispanic blacks accounted for the largest proportion of not meeting AI compared to their comparts, respectively (Figure 3B1,B3; Table S1).

The trend of sodium was investigated by exceeding UL with increased trend from 77.0% to 78.9% (*p*_for linear trend_ = 0.002; Table 3), which was apparent in men aged ≥ 46 years, women aged ≥ 46 years, participants with low and medium socioeconomic status, Hispanics, non-Hispanic whites, non-Hispanic blacks, underweight, and normal weight, (*p*_for linear trend_ = 0.001, 0.007, 0.021, 0.018, 0.006, 0.015, 0.013, 0.004, and 0.002, respectively) (Figure 3A1–A4). Men aged 19–45 years and participants with high socioeconomic status accounted for the largest proportion of exceeding UL compared to their comparts, respectively (Figure 3A1,A2; Table S1).

### 3.3. Trends of Vitamins Not Meeting DRIs

Trends of vitamins were estimated by not meeting RDA except for vitamin K by not meeting AI, with decreased trends for 4 kinds of vitamins, being vitamin B_6_, E, K, and choline (*p*_for linear trend_ = 0.005, < 0.001, < 0.001, and = 0.011, respectively); increased trends for 5 kinds of vitamins, being vitamin B_1_, B_2_, B_12_, C, and folate DFE (*p*_for linear trend_ = 0.018, 0.046, < 0.001, = 0.001, and 0.003, respectively); and stable trends for 3 kinds of vitamins, being vitamin A, B_3_, and D (*p*_for linear trend_ = 0.544, 0.120, and 0.213, respectively). The decreased trends were from 34.0% to 30.6%, 94.6% to 88.5%, 74.6% to 62.1%, 87.2% to 85.5%, and the increased trends were from 26.2% to 29.6%, 12.0% to 14.4%, 20.6% to 24.0%, 58.9% to 64.1%, and 37.0% to 42.1% for the above vitamins, respectively (Table 3).

The increased trends were observed in intakes of vitamin B_3_, B_6_, and E, being from 24.69 to 25.80 mg/day, 1.91 to 2.11 mg/day, and 7.05 to 9.14 mg/day, (*p*_for linear trend_ = 0.002, < 0.001, and < 0.001, respectively), while the decreased trends were observed in intakes of vitamin B_1_, B_2_, and C, being from 1.68 to 1.58 mg/day, 2.27 to 2.16 mg/day, and 87.83 to 78.32 mg/day (*p*_for linear trend_ < 0.001, = 0.002 and < 0.001, respectively). On the other hand, the decreased trend was observed in folate DFE from 2003 to 2012 (545.98 to 559.13 mg/day), but increased slightly thereafter (Table 2). 

**Figure 4:**
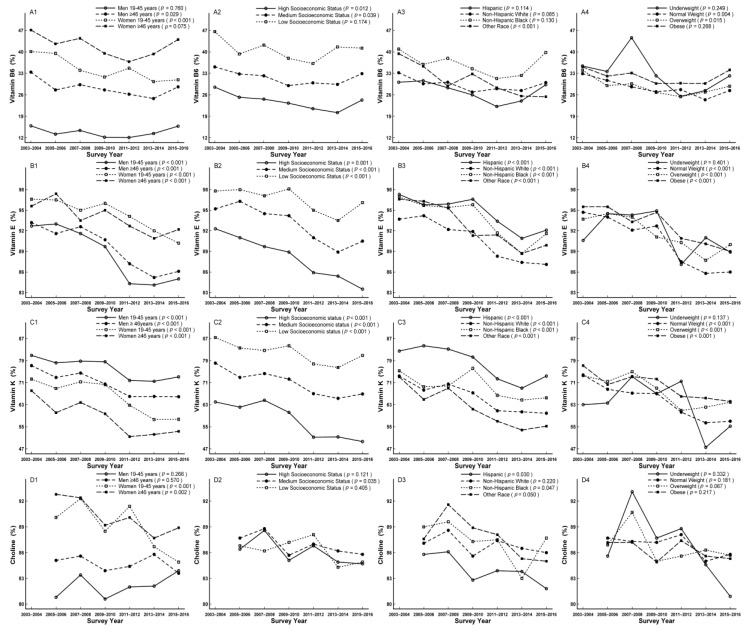
Trends of dietary vitamin B6 (**A1**–**A4**), vitamin E (**B1**–**B4**), vitamin K (**C1**–**C4**), and choline (**D1**–**D4**) by sex, age, socioeconomic status, race/ethnicity, and body mass index (BMI) for percentage not meeting dietary reference intakes among US adults across seven surveys in the National Health and Nutrition Examination Survey (NHANES 2003–2016).

**Figure 5:**
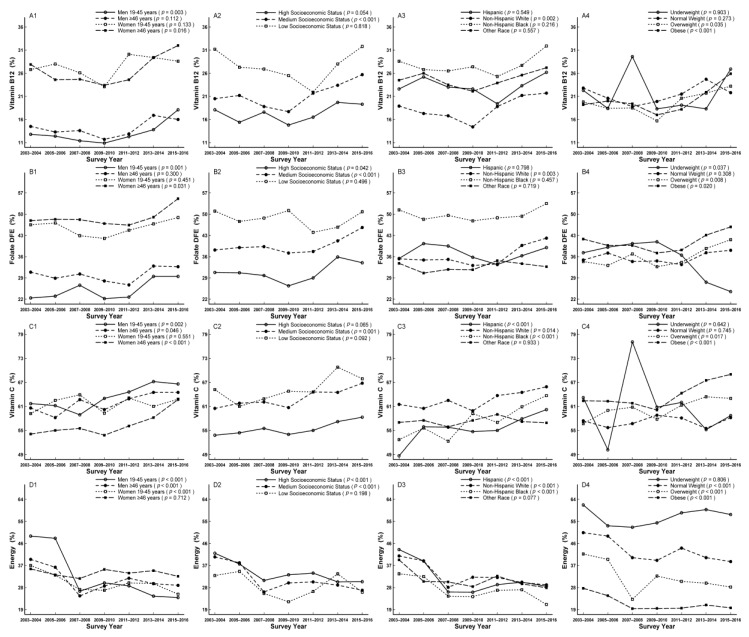
Trends of dietary vitamin B12 (**A1**–**A4**), folate DFE (**B1**–**B4**), vitamin C (**C1**–**C4**), and energy (**D1**–**D4**) by sex, age, socioeconomic status, race/ethnicity, and body mass index (BMI) for percentage not meeting dietary reference intakes among US adults across seven surveys in the National Health and Nutrition Examination Survey (NHANES 2003–2016).

In subgroup analysis, the decreased trends were observed in men aged ≥46 years, women aged 19–45 years, participants with high and medium socioeconomic status, other race, normal weight, and overweight for vitamin B_6_ (*p*_for linear trend_ = 0.029, < 0.001, = 0.012, 0.039, < 0.001, = 0.004, and 0.015, respectively) (Figure 4A1–A4), in women, participants with medium socioeconomic status, Hispanics, and non-Hispanic blacks for choline (*p*_for linear trend_ < 0.001, = 0.002, 0.035, 0.030, and 0.047, respectively) (Figure 4D1–D4), in all subgroups except for underweight for vitamin E and K (*p*_for linear trend_ = 0.401, and 0.137, respectively) (Figure 4B1–B4,C1–C4), and in underweight for individual folate DFE (*p*_for linear trend_ = 0.037) (Figure 5B–4). The increased trends were apparent in men aged 19–45 years, women aged ≥ 46 years, participants with medium socioeconomic status, non-Hispanic whites, overweight, and obese for vitamin B_12_ (*p*_for linear trend_ = 0.003, 0.016, < 0.001, = 0.002, 0.035, and < 0.001, respectively; Figure 5A1–A4), in men aged 19–45 years, women aged ≥ 46 years, participants with medium and high socioeconomic status, non-Hispanic whites, underweight, overweight, and obese for folate DFE (*p*_for linear trend_ = 0.001, 0.031, 0.042, < 0.001, = 0.003, 0.037, 0.008, and 0.020, respectively; Figure 5B1–B4), in men, women aged ≥ 46 years, participants with medium socioeconomic status, Hispanics, non-Hispanic whites, non-Hispanic blacks, overweight, and obese for vitamin C (*p*_for linear trend_ = 0.002, 0.046, < 0.001, = 0.001, < 0.001, = 0.014, < 0.001, = 0.017, and < 0.001, respectively; Figure 5C1–C4). 

In addition, participants with low socioeconomic status for vitamin B_6_, B_12_, C, E, K, and folate DFE, women aged ≥ 46 years, and non-Hispanic blacks for vitamin B_6_, men aged 19–45 years and Hispanics for vitamin K, women and non-Hispanic blacks for vitamin B_12_ and folate DFE, and non-Hispanic whites for vitamin C accounted for the largest proportion of not meeting RDA or AI compared to their comparts, respectively (Figure 4 and Figure 5, Table S1).

### 3.4. Trends of Energy Intake Exceeding EER

The significant decreased trends were observed in both energy intake and percentage of exceeding EER, from 2189.45 to 2077.27 kcal/day and from 40.4% to 28.0% (*p*_for linear trend_ = 0.001 and < 0.001, respectively; Table 2 and Table 3). In subgroups analysis, the decreased trends for exceeding EER were not observed in women aged ≥ 46 years, low socioeconomic status, other race, and underweight (*p*_for linear trend_ = 0.712, 0.198, 0.077, and 0.806, respectively; Figure 5D1–D4). Participants with underweight accounted for the largest proportion while the obese accounted for the smallest proportion of exceeding EER compared to their comparts (Figure 5D4, Table S1).

### 3.5. Trends of Other Dietary Nutrients

There were significant increased trends in intakes of alpha-carotene and beta-carotene from 2003 to 2012 (384.36 to 497.44 µg/day and 2064.80 to 2561.42 µg/day, respectively), but slightly decreased thereafter. There were significant increased trends in intake of lutein + zeaxanthin, from 1456.81 to 1633.81 µg/day (*p*_for linear trend_ = 0.001, Table 2). In subgroups analysis, these increased trends were observed in men aged ≥ 46 years, other race, and the obese for alpha-carotene (*p*_for linear trend_ = 0.044, 0.047, and 0.017, respectively), and in women aged 19–46 years, participants with medium socioeconomic status, non-Hispanic whites, non-Hispanic blacks, other race, the underweight, and the obese for beta-carotene (*p*_for linear trend_ = 0.001, 0.001, 0.038, 0.007, 0.004, < 0.001, and = 0.010, respectively; Table S2). The highest intakes of alpha-carotene and beta-carotene were consistently observed in participants with high socioeconomic status and other race compared to other participants, respectively (Figure S2A2,A3,B2,B3; Table S2). The lowest intake of alpha-carotene was observed in non-Hispanic blacks, while beta-carotene was observed in Hispanics, respectively (Figure S2A3,B3; Table S2). The lower beta-carotene intake was observed in men and women aged 19–45 years (Figure S2B1, Table S2).

The significantly decreased trends were observed in intakes of beta-cryptoxanthin, lycopene, caffeine and theobromine, from 141.72 to 86.66 µg/day, from 6535.77 to 5301.69 µg/day, from 179.51 to 166.78 mg/day, and from 41.75 to 32.44 mg/day (*p*_for linear trend_ < 0.001, < 0.001, = 0.004, and < 0.001), respectively; Table 2). These decreased trends were apparent for lycopene in participants aged 19–45 years, participants with high and medium socioeconomic status, Hispanics, non-Hispanic whites, the overweight, and the obese (*p*_for linear trend_ < 0.001, < 0.001, = 0.003, < 0.001, 0.014, < 0.001, < 0.001, and = 0.003, respectively), and caffeine in participants aged 19–45 years, participants with medium socioeconomic status, non-Hispanic whites, non-Hispanic blacks, other race, normal weight, and the overweight (*p*_for linear trend_ < 0.001, < 0.001, = 0.002, 0.036, 0.041, 0.002, 0.008, and 0.003, respectively) (Table S2). In subgroup analysis, the highest lycopene intake was found in men aged ≥ 46 years and participants with high socioeconomic status compared to other participants (Figure S2D1,D2; Table S2). In addition, the highest intake of caffeine was observed in men aged ≥ 46 years, participants with high socioeconomic status, and non-Hispanic whites, while the lowest intake in women aged 19–45 years, participants with low socioeconomic status, and non-Hispanic blacks, respectively (Figure S2D1–D4, Table S2).

### 3.6. Sensitivity Analysis

When the analysis was limited to participants who did not have implausible energy intake, most of the above results were unchanged (Tables S3 and S4). A few previous non-significant trends turned to be statistically significant trends, including increased trends were found in calories from protein, total fat, saturated fatty acids, and polyunsaturated fatty acids, 16.6% to 17.5% (*p*_for linear trend_ = 0.004), 36.4% to 39.3% (*p*_for linear trend_ < 0.001), 12.6% to 13.5% (*p*_for linear trend_ = 0.007), and 8.2% to 9.5% (*p*_for linear trend_ < 0.001), respectively, and a decreased trend was found in calories from total sugars being from 24.3% to 22.4% (*p*_for linear trend_ < 0.001). And the increased trend for potassium not meeting AI was found to be non-significant.

## 4. Discussion

Based on this large nationally representative survey of US adults across 14 years (2003–2016), many dietary nutrients were improved but some nutrients are still needed to be better.

Increased trends of meeting DRIs were observed for dietary carbohydrate and fiber, which indicated that not only dietary carbohydrate but also one of its important compositions were improved. However, there were still some improvements that needed to be made. All adults should be recommended to increase both carbohydrate and fiber intake, since their percentages of meeting DRIs were relatively small (33.2% and 11.1%, respectively) across these years, especially for men, low socioeconomic status, and non-Hispanic blacks. In addition, total sugars need to be eaten less because of its increasing trend across these years especially for women, with 94.7% of them exceeding the DGA.

Although respective increased and decreased trends were observed in dietary polyunsaturated fatty acids and monounsaturated fatty acids, no statistically significant trend was found for dietary total fat and thus, dietary fat and its compositions should be improved. Giving the evidence that the average percentage of calories from saturated fatty acids was 13.3%, which was higher than the recommendation of 10% in the 2015 Dietary Guidelines for Americans and that of 12.7% in the National Health Interview Survey (NHIS) in 1992 [9], dietary saturated fatty acids should be replaced with monounsaturated and polyunsaturated fatty acids, especially for women aged ≥46 years with the value of 15.9%.

Stable trends were observed for protein on both the percentages of not meeting DRIs and of calories from it during the present 7 surveys. The average of calories from protein was 17.9% in the present analysis, which seemed to be similar with that of 17.0% in the NHANES I (1971 to 1975) [15]. Although the average of calories from protein was in the range of the AMDR, 27.4% of men aged 19–45 years should take care of their protein intake based on the fact that their calories from protein did not meet the AMDR. 

For minerals, large percentages of US adults were observed meeting the DRIs for phosphorus and selenium, which needs to be maintained. In contrast, no improvements were observed for iron, magnesium, calcium, sodium, and potassium across this 14-year period, whereas some of them worsened. Thus, reasonable intakes of the above minerals should be considered seriously. For certain items, women aged 19–45 years should pay much more attention to increasing their dietary iron intake, based on 81.7% of them not meeting the RDA, which was much higher than the 6.7% for men aged 19–45 years. Eating more legumes, seafood, meats, and poultry for these women might benefit in helping them meeting the RDA for dietary iron [16]. In addition, the averages of dietary calcium, magnesium, zinc and copper not meeting the RDA were notably higher among participants with low socioeconomic status. It has been reported that socioeconomic status is closely related to dietary intake, and the gap between higher and lower socioeconomic status has expanded over time [17]. To improve diet for participants with low socioeconomic status, it might be helpful that the government and nongovernmental organizations should try their best to allocate a certain amount of funds and educated them in nutrition health [18,19]. In addition, the percentages of not meeting DRIs were increased for sodium and potassium. The average of dietary sodium was 3495.32 mg/day across recent 14 years which was much higher than the UL of 2300 mg/day [14]. Although the Dietary Guidelines for Americans, as well as other public health organizations have always made initiative efforts, reducing sodium intake is still the most important challenge [20,21], especially for men and participants with high socioeconomic status who contributed a large proportion of exceeding UL. The most likely reason might be that men tend to consume more sodium owing to the consumption of more foods than women and people with high socioeconomic status prefer to go to restaurants and buy processed meat products to eat [22,23]. It has been reported that most dietary sodium comes from salt added in commercial food processing and restaurants while only a small proportion comes from the sodium inherent in food or salt added to home cooking [24]. Therefore, to reduce sodium, it might be useful to help people know the sodium information on food labels or restaurant menus by providing individual health education, and reformulating food to reduce sodium content in retail and food service agencies [25,26,27,28]. For potassium, based on the majority of US adults with inadequate potassium intake especially for women (98.7%) and non-Hispanic blacks (97.1%), great effort should be made to increase dietary potassium by improving the food environment and encouraging adults to take more fresh fruits and vegetables, which are usually good sources of potassium [14].

For vitamins, our findings suggested that vitamin B_6_ and choline contributed to vitamin improvements. Compared to estimated intakes below their respective RDA for vitamin B_6_ which were 71% for men and 90% for women in NHANES II [5], the present results showed that they were remarkably decreased to 21.0% and 38.2%, respectively. However, other vitamins need to be improved. Increased trends of not meeting DRIs for dietary vitamin B_12_ and folate DFE were observed, especially for all women, which could be improved by recommending that they consume foods fortified with vitamin B_12_ and folate DFE, such as seafood, dairy products, beans and peas, oranges, and dark-green leafy vegetables [29,30]. In addition, more than 50% of US adults were observed not meeting the RDA for dietary vitamin C, D, E, and K. Compared to the NHIS in 1992 with vitamin E of 8.40 mg/day and the NHANES I Epidemiologic Follow-up Study (NHEFS) from 1971 to 1975 with vitamin C of 82.0 mg/day [9,31], the present results of, respectively, 8.19 and 84.0 mg/day were not changed much. All adults should be encouraged to eat vitamin-rich foods to increase these vitamin levels, such as fruits, seafood, nutsy, seeds, and dark-green vegetables [32], especially for participants with low socioeconomic status who contributed to the largest proportion of not meeting RDA or AI.

Epidemiological and clinical data also addressed the importance of the common carotenoids in the daily life, which were alpha-carotene, beta-carotene and lutein + zeaxanthin, beta-cryptoxanthin, and lycopene [33,34,35]. Based on the relatively low intake levels compared to their comparts, adults aged 19–45 years and Hispanics should eat more food rich in beta-carotene, women and non-Hispanic blacks should eat more food rich in lycopene, and people with low socioeconomic status should eat more food rich in both beta-carotene and lycopene, such as tomato and guava [36]. As for dietary caffeine, moderate coffee consumption up to 400 mg/day of caffeine has been suggested as a healthy eating pattern [14]. Our research showed a significant decreased trend for caffeine, but there were still 9.3% of people who consumed more than 400 mg/day that should consider limiting their caffeine intake. 

A low-calorie healthy diet may provide a possible solution to the ongoing challenge of preventing and controlling obesity and cardiovascular diseases [37]. Although the average energy intake being 2127.0 kcal/day in the present study was a little higher than the 1971.8 kcal/day in NHANES I (1971–1975) or similar to 2233.9 kcal/day in NHANES III (1988–1994) [15], significant decreased trends for both energy intake and percentage of exceeding EER were found in the present study, especially for participants being normal weight, overweight and obese. These improvements might be attributed by the fact that 7.1% of the overweight and 12.7% of the obese participants were trying to lose weight and be healthy by eating less.

Possible limitations in the present study should be noted: dietary nutrients were collected based on two days of 24-h dietary recall survey, which may lead a uniform underestimation or overestimation of actual dietary intake. In addition, recall bias cannot be excluded when assessing sources of dietary intake.

## 5. Conclusions

In summary, the dietary nutrients of US adults have improved from 2003 to 2016 but are still far from the optimal target for all adults. Across the entire population, further improvements are needed for dietary carbohydrate, total fat, total sugars, fiber, saturated fatty acids, calcium, magnesium, potassium, sodium, vitamin A, vitamin C, vitamin D, vitamin E, and vitamin K. People with low socioeconomic status and non-Hispanic blacks should be focused on to make them meet the DRIs of most of the nutrients. These findings could help health professionals and policy makers design effective strategies to improve the American diet.

## Figures and Tables

**Table 1:** Percentages of US adults by population characteristics in the National Health and Nutrition Examination Survey (NHANES 2003–2016).

	No. of Participants (%)						
	2003–2004	2005–2006	2007–2008	2009–2010	2011–2012	2013–2014	2015–2016
All	4354	4330	5265	5586	4713	4972	4879
Sex Age							
Men 19–45 y	1039 (23.9)	1091 (25.2)	1160 (22.0)	1226 (21.9)	1143 (24.3)	1128 (22.7)	1060 (21.7)
Men 46+ y	1159 (26.6)	1128 (26.1)	1467 (27.9)	1516 (27.1)	1223 (25.9)	1264 (25.4)	1319 (27.0)
Women 19–45 y	953 (21.9)	1046 (24.2)	1150 (21.8)	1335 (23.9)	1084 (23.0)	1192 (24.0)	1123 (23.0)
Women 46+ y	1203 (27.6)	1065 (24.6)	1488 (28.3)	1509 (27.0)	1263 (26.8)	1388 (27.9)	1377 (28.2)
Socioeconomic status ^1^							
High	904 (20.8)	1046 (24.2)	1092 (20.7)	1132 (20.3)	1098 (23.3)	1211 (24.4)	1022 (20.9)
Medium	2853 (65.5)	2756 (63.6)	3401 (64.6)	3684 (66.0)	3033 (64.4)	3182 (64.0)	3296 (67.6)
Low	597 (13.7)	528 (12.2)	772 (14.7)	770 (13.8)	582 (12.3)	579 (11.6)	561 (11.5)
Race/ethnicity							
Hispanic	988 (22.7)	979 (22.6)	1437 (27.3)	1513 (27.1)	888 (18.8)	1084 (21.8)	1459 (29.9)
Non-Hispanic white	2309 (53.0)	2150 (49.7)	2508 (47.6)	2750 (49.2)	1816 (38.5)	2217 (44.6)	1681 (34.5)
Non-Hispanic black	890 (20.4)	1028 (23.7)	1113 (21.1)	1030 (18.4)	1269 (26.9)	996 (20.0)	1036 (21.2)
Other race	167 (3.8)	173 (4.0)	207 (3.9)	293 (5.2)	740 (15.7)	675 (13.6)	703 (14.4)
BMI ^2^							
Underweight	74 (1.7)	73 (1.7)	93 (1.7)	92 (1.6)	96 (2.0)	87 (1.7)	69 (1.4)
Normal weight	1361 (31.3)	1307 (30.2)	1473 (28.0)	1516 (27.1)	1422 (30.2)	1421 (28.6)	1264 (25.9)
Overweight	1512 (34.7)	1457 (33.6)	1810 (34.4)	1858 (33.3)	1490 (31.6)	1587 (31.9)	1561 (32.0)
Obese	1407 (32.3)	1493 (34.9)	1889 (35.9)	2120 (38.0)	1705 (36.2)	1877 (37.8)	1905 (40.7)

y, years. ^1^ Socioeconomic status were defined based on educational attainment (EA) and a poverty income ratio (PIR), participants were classified into high (more than 12 completed years of EA and a PIR of at least 3.5), low (less than 12 years of EA and a PIR less than 1.30), and medium (others). ^2^ Body mass index (BMI) was defined as weight divided by height squared and participants were classified into underweight (<18.5 kg/m^2^), normal (18.5–24.9 kg/m^2^), overweight (25.0–29.9 kg/m^2^), and obese (≥30 kg/m^2^).

**Table 2:** Trends in dietary nutrient intake among US adults in the National Health and Nutrition Examination Survey (NHANES 2003–2016) ^1.^

	2003–2004	2005–2006	2007–2008	2009–2010	2011–2012	2013–2014	2015–2016	*p*_for linear trend_^2^	2003–2016
Macro-nutrients									
Energy (kcal/day)	2189.45 ± 16.35	2148.31 ± 24.17	2117.81 ± 21.41	2126.45 ± 23.44	2139.51 ± 16.80	2080.30 ± 15.53	2077.27 ± 20.61	<0.001	2127.00 ± 7.49
Protein (g/day)	83.82 ± 1.07	84.08 ± 0.84	82.46 ± 0.85	83.30 ± 0.93	82.52 ± 0.61	83.48 ± 0.65	81.90 ± 0.77	0.129	83.06 ± 0.31
Carbohydrate (g/day)	265.14 ± 2.35	258.26 ± 2.85	256.73 ± 2.74	258.26 ± 2.56	260.05 ± 2.25	248.04 ± 2.04	241.08 ± 2.55	<0.001	255.12 ± 0.94
Total fat (g/day)	82.87 ± 0.78	81.58 ± 1.23	80.53 ± 1.07	79.39 ± 1.06	80.61 ± 1.00	81.08 ± 0.71	82.92 ± 0.99	0.968	81.28 ± 0.38
Total sugars (g/day)	122.59 ± 1.54	117.65 ± 1.77	116.57 ± 2.40	115.42 ± 1.33	114.32 ± 1.43	108.51 ± 1.36	102.94 ± 1.63	<0.001	113.79 ± 0.65
Dietary fiber (g/day)	15.67 ± 0.37	15.83 ± 0.21	16.17 ± 0.37	17.11 ± 0.21	17.87 ± 0.26	17.03 ± 0.20	17.33 ± 0.37	<0.001	16.74 ± 0.12
Saturated fatty acids (g/day)	27.31 ± 0.30	27.24 ± 0.40	26.58 ± 0.42	25.49 ± 0.42	25.98 ± 0.42	26.27 ± 0.23	27.21 ± 0.38	0.167	26.64 ± 0.14
Monounsaturated fatty acids (g/day)	31.10 ± 0.31	29.99 ± 0.44	29.74 ± 0.40	28.52 ± 0.39	28.79 ± 0.36	28.28 ± 0.26	29.12 ± 0.36	<0.001	29.33 ± 0.14
Polyunsaturated fatty acids (g/day)	17.47 ± 0.19	17.30 ± 0.33	17.22 ± 0.22	17.70 ± 0.21	19.24 ± 0.23	18.85 ± 0.23	19.04 ± 0.24	<0.001	18.14 ± 0.09
Cholesterol (g/day)	293.08 ± 5.39	289.57 ± 3.75	292.16 ± 4.67	278.22 ± 4.68	281.89 ± 3.40	293.15 ± 3.80	298.29 ± 4.93	0.546	289.51 ± 1.69
Minerals									
Calcium (mg/day)	880.28 ± 16.93	931.71 ± 12.98	934.41 ± 17.68	1011.61 ± 10.92	982.95 ± 12.81	969.20 ± 9.13	954.03 ± 18.33	<0.001	952.93 ± 5.51
Phosphorus (mg/day)	1335.34 ± 17.91	1338.62 ± 12.90	1336.79 ± 17.29	1412.72 ± 14.02	1404.13 ± 11.98	1395.84 ± 11.84	1379.40 ± 18.44	<0.001	1372.64 ± 5.69
Magnesium (mg/day)	279.88 ± 4.76	297.93 ± 2.87	293.42 ± 5.30	305.19 ± 3.14	307.12 ± 3.91	302.42 ± 3.42	304.25 ± 4.59	<0.001	298.84 ± 1.60
Iron (mg/day)	16.07 ± 0.22	16.21 ± 0.14	15.55 ± 0.29	15.63 ± 0.12	15.43 ± 0.11	14.63 ± 0.12	14.03 ± 0.17	<0.001	15.34 ± 0.07
Zinc (mg/day)	12.24 ± 0.22	12.67 ± 0.20	12.17 ± 0.22	12.04 ± 0.16	11.35 ± 0.13	11.24 ± 0.08	11.20 ± 0.15	<0.001	11.83 ± 0.07
Copper (mg/day)	1.27 ± 0.02	1.39 ± 0.01	1.34 ± 0.02	1.32 ± 0.01	1.30 ± 0.02	1.22 ± 0.01	1.25 ± 0.02	<0.001	1.30 ± 0.01
Sodium (mg/day)	3482.76 ± 32.48	3482.98 ± 38.03	3452.07 ± 42.64	3588.35 ± 33.23	3544.16 ± 27.54	3499.96 ± 22.59	3501.33 ± 34.88	0.323	3507.86 ± 12.61
Potassium (mg/day)	2722.75 ± 33.32	2706.15 ± 26.25	2653.48 ± 36.98	2763.75 ± 27.61	2754.59 ± 34.18	2647.47 ± 29.15	2634.85 ± 33.13	0.086	2696.81 ± 11.89
Selenium (µg/day)	111.24 ± 1.42	111.35 ± 1.39	111.77 ± 1.22	113.70 ± 1.32	114.86 ± 1.11	117.35 ± 0.95	115.34 ± 1.15	<0.001	113.73 ± 0.47
Vitamins									
Vitamin A (µg/day)	614.12 ± 13.28	635.36 ± 9.33	632.65 ± 17.75	654.41 ± 12.14	664.99 ± 27.07	640.34 ± 12.63	636.44 ± 10.53	0.098	640.06 ± 5.98
Vitamin B_1_ (mg/day)	1.68 ± 0.02	1.68 ± 0.01	1.65 ± 0.03	1.68 ± 0.01	1.63 ± 0.01	1.63 ± 0.02	1.58 ± 0.02	<0.001	1.65 ± 0.01
Vitamin B_2_(mg/day)	2.27 ± 0.04	2.26 ± 0.02	2.22 ± 0.04	2.17 ± 0.03	2.13 ± 0.03	2.18 ± 0.03	2.16 ± 0.03	0.002	2.20 ± 0.01
Vitamin B_3_ (mg/day)	24.69 ± 0.34	25.77 ± 0.23	25.46 ± 0.34	26.20 ± 0.22	25.83 ± 0.28	26.56 ± 0.23	25.80 ± 0.29	0.002	25.77 ± 0.11
Vitamin B_6_ (mg/day)	1.91 ± 0.03	2.04 ± 0.02	2.03 ± 0.03	2.14 ± 0.01	2.16 ± 0.03	2.23 ± 0.03	2.11 ± 0.03	<0.001	2.09 ± 0.01
Vitamin B_12_ (µg/day)	5.34 ± 0.17	5.66 ± 0.13	5.50 ± 0.14	5.44 ± 0.07	5.21 ± 0.12	5.02 ± 0.07	5.06 ± 0.14	0.002	5.30 ± 0.05
Folate DFE (µg/day)	545.98 ± 9.71	549.10 ± 7.00	549.69 ± 13.16	556.54 ± 4.99	559.13 ± 5.65	528.22 ± 7.03	515.96 ± 8.71	0.006	541.91 ± 3.12
Vitamin C (mg/day)	87.83 ± 2.89	87.52 ± 1.72	85.15 ± 3.47	86.28 ± 1.43	84.33 ± 3.39	79.13 ± 1.50	78.32 ± 2.18	<0.001	84.00 ± 0.91
Vitamin D (µg/day)	NA	NA	4.41 ± 0.09	5.14 ± 0.10	4.66 ± 0.11	4.79 ± 0.11	4.59 ± 0.10	0.973	4.72 ± 0.04
Vitamin E (mg/day)	7.05 ± 0.10	7.32 ± 0.11	7.61 ± 0.17	7.98 ± 0.09	8.81 ± 0.14	9.23 ± 0.16	9.14 ± 0.20	<0.001	8.19 ± 0.06
Vitamin K (µg/day)	94.76 ± 2.06	102.45 ± 3.44	98.57 ± 3.99	104.49 ± 2.33	132.53 ± 11.54	119.34 ± 2.86	119.55 ± 3.48	0.092	110.59 ± 2.05
Choline (mg/day)	NA	331.37 ± 2.76	324.84 ± 4.21	338.88 ± 4.56	331.06 ± 3.46	336.50 ± 3.88	335.98 ± 3.65	0.088	333.18 ± 1.55
Alpha-carotene (µg/day)	384.36 ± 16.66	382.54 ± 17.58	403.69 ± 26.39	438.66 ± 17.71	497.44 ± 49.08	419.71 ± 20.37	409.56 ± 26.23	0.034	420.01 ± 10.43
Beta-carotene (µg/day)	2064.80 ± 71.57	2026.06 ± 65.44	2076.97 ± 103.97	2244.24 ± 60.66	2561.42 ± 183.22	2287.28 ± 85.81	2219.79 ± 73.79	0.007	2214.97 ± 38.90
Beta-cryptoxanthin (µg/day)	141.72 ± 7.10	133.44 ± 4.96	77.28 ± 5.15	87.09 ± 4.48	81.12 ± 2.30	83.96 ± 2.78	86.66 ± 4.71	<0.001	98.04 ± 1.76
Lycopene (µg/day)	6535.77 ± 360.40	5465.93 ± 149.11	5772.08 ± 136.63	5526.56 ± 220.14	5301.55 ± 195.18	4948.96 ± 104.30	5301.69 ± 163.12	<0.001	5536.28 ± 73.08
Lutein + zeaxanthin (µg/day)	1456.81 ± 57.89	1426.52 ± 49.92	1409.49 ± 69.36	1534.59 ± 53.95	1785.77 ± 109.23	1644.09 ± 56.81	1633.81 ± 63.41	0.001	1559.04 ± 26.66
Caffeine (mg/day)	179.51 ± 7.30	179.80 ± 5.67	178.94 ± 8.62	170.02 ± 7.06	165.10 ± 7.11	159.56 ± 5.13	166.78 ± 4.63	0.004	171.16 ± 2.51
Theobromine (mg/day)	41.75 ± 1.53	37.73 ± 1.44	38.77 ± 1.54	38.30 ± 1.00	35.90 ± 1.75	37.47 ± 1.18	32.44 ± 1.07	<0.001	37.39 ± 0.53
Percentage of energy									
Protein (%, kcal)	17.2%	17.8%	18.4%	17.8%	17.5%	18.4%	18.3%	0.266	17.9%
Carbohydrate (%, kcal)	53.9%	54.6%	57.1%	54.7%	54.4%	54.6%	53.7%	0.356	54.7%
Total fat (%, kcal)	37.5%	38.1%	39.9%	37.3%	37.4%	39.7%	41.0%	0.273	38.7%
Total Sugars (%, kcal)	25.0%	24.9%	26.0%	24.5%	24.1%	24.1%	23.2%	0.109	24.5%
Saturated fatty acids (%, kcal)	12.9%	13.3%	13.8%	12.7%	12.6%	13.4%	14.0%	0.322	13.3%
Monounsaturated fatty acids (%, kcal)	14.3%	14.3%	15.0%	13.7%	13.6%	14.2%	14.8%	0.919	14.3%
Polyunsaturated fatty acids (%, kcal)	8.5%	8.7%	9.2%	8.9%	9.4%	9.7%	9.9%	0.191	9.2%

NA, not available. DFE, dietary folate equivalents. ^1^ Values are weighted means ± standard error (SE), determined with adjustment for the complex sampling design of NHANES. ^2^ *p* for linear trend for the overall F test for each variable from survey-weighted linear regression.

**Table 3:** Trends in percentage of dietary nutrient intake not meeting dietary reference intakes among US adults in the National Health and Nutrition Examination Survey (NHANES 2003–2016).

	2003–2004	2005–2006	2007–2008	2009–2010	2011–2012	2013–2014	2015–2016	*p*_for linear trend_^1^	2003–2016
Macro-Nutrients									
Energy (kcal/day) *****	40.4%	37.7%	27.6%	30.3%	31.2%	29.8%	28.0%	<0.001	32.0%
Protein (kcal/day) ***	21.2%	20.7%	19.8%	19.0%	20.5%	19.8%	19.8%	0.219	20.1%
Carbohydrate (kcal/day) ***	68.4%	68.9%	67.5%	65.7%	65.4%	66.2%	66.1%	0.001	66.8%
Total Fat (kcal/day) ***	74.4%	73.1%	73.6%	71.7%	69.5%	73.0%	73.2%	0.128	72.6%
Total Sugars (kcal/day) ****	87.5%	87.1%	88.3%	87.2%	86.6%	86.8%	85.3%	0.047	86.9%
Dietary fiber (g/day) *	92.0%	91.7%	90.3%	87.8%	85.6%	88.0%	87.1%	<0.001	88.9%
Saturated fatty acids(kcal/day) ****	62.8%	65.1%	67.6%	64.2%	61.8%	67.5%	70.6%	<0.001	65.6%
Minerals									
Calcium (mg/day)	70.2%	66.5%	67.7%	61.2%	62.5%	64.5%	64.5%	<0.001	65.2%
Iron (mg/day)	31.2%	29.7%	30.5%	30.6%	29.7%	32.7%	34.5%	0.006	31.3%
Magnesium (mg/day)	81.1%	76.2%	77.0%	74.5%	72.7%	75.0%	74.4%	<0.001	75.8%
Phosphorus(mg/day)	9.8%	9.6%	9.1%	6.6%	7.5%	7.6%	8.6%	0.011	8.4%
Potassium (mg/day) *	94.4%	94.7%	95.7%	94.9%	95.2%	95.4%	96.1%	0.006	95.2%
Sodium (mg/day) **	77.0%	77.1%	76.7%	81.2%	80.8%	80.4%	78.9%	0.002	78.9%
Zinc (mg/day)	35.6%	36.3%	38.1%	36.9%	39.9%	41.4%	41.5%	<0.001	38.6%
Copper (mg/day)	28.8%	22.7%	25.1%	24.9%	28.4%	30.9%	30.7%	<0.001	27.4%
Selenium (µg/day)	9.8%	10.0%	10.4%	8.2%	8.3%	8.2%	8.2%	<0.001	9.0%
Vitamins									
Vitamin A (µg/day)	75.7%	73.5%	72.6%	72.9%	72.6%	72.7%	74.8%	0.544	73.5%
Vitamin B_1_ (mg/day)	26.2%	26.6%	27.2%	24.5%	27.0%	27.8%	29.6%	0.018	27.0%
Vitamin B_2_(mg/day)	12.0%	12.7%	13.2%	14.2%	14.6%	13.7%	14.4%	0.046	13.6%
Vitamin B_3_ (mg/day)	17.2%	15.8%	16.7%	13.7%	14.3%	14.1%	16.4%	0.120	15.4%
Vitamin B_6_(mg/day)	34.0%	30.7%	30.7%	28.0%	27.7%	27.3%	30.6%	0.005	29.8%
Vitamin B_12_ (µg/day)	20.6%	19.7%	19.1%	17.4%	20.0%	22.5%	24.0%	<0.001	20.5%
Folate DFE (µg/day)	37.0%	36.8%	37.1%	35.0%	35.4%	39.8%	42.1%	0.003	37.7%
Vitamin C (mg/day)	58.9%	59.3%	60.1%	59.0%	61.4%	62.6%	64.1%	0.001	60.8%
Vitamin D (µg/day)	NA	NA	97.5%	96.2%	97.2%	95.9%	96.9%	0.213	96.7%
Vitamin E (mg/day)	94.6%	94.7%	93.2%	92.9%	89.6%	88.1%	88.5%	<0.001	91.6%
Vitamin K (µg/day) *	74.6%	70.0%	72.0%	69.6%	62.7%	61.6%	62.1%	<0.001	67.3%
Choline (mg/day)	NA	87.2%	88.5%	85.6%	87.0%	85.6%	85.5%	0.011	86.6%

NA, not available. DFE, dietary folate equivalents. ^1^ *p* *for linear* trend for the overall F test for each variable from survey-weighted linear regression. * Not meeting adequate intakes (AI). ** Exceeding upper intake level (UL). *** Not meeting acceptable macronutrient distribution range (AMDR). **** Not meeting 2015–2020 Dietary Guidelines recommended limit (DGA). Values are means, determined with adjustment for the complex sampling design of NHANES.

## Supplementary Materials

The following are available online at https://www.mdpi.com/2072-6643/11/11/2617/s1, Figure S1: Flowchart, Figure S2: Trends for dietary alpha-carotene (A1–A4), beta-carotene (B1–B4), lycopene (C1–C4), and caffeine (D1–D4) intake by age sex, socioeconomic status, race/ethnicity, and body mass index (BMI) in US adults across seven surveys in the National Health and Nutrition Examination Survey (NHANES 2003–2016), Table S1: Trends in percentage of dietary nutrient intake not meeting Dietary Reference Intakes by age sex, socioeconomic status, race/ethnicity, and BMI among US adults in the National Health and Nutrition Examination Survey (NHANES 2003–2016), Table S2: Intake of increased and decreased nutrients for US adults across seven surveys by age sex, socioeconomic status, race/ethnicity, and BMI in the National Health and Nutrition Examination Survey (NHANES 2003–2016).

## References

[1] World Health Organization (2003). Diet, Nutrition and the Prevention of Chronic Diseases.

[2] Mokdad A.H., Ballestros K., Echko M., Glenn S., Olsen H.E., Mullany E., Lee A., Khan A.R., Ahmadi A., US Burden of Disease Collaborators (2018). The State of US Health, 1990–2016: Burden of Diseases, Injuries, and Risk Factors among US States. JAMA.

[3] World Health Organization (1990). Diet, Nutrition, and the Prevention of Chronic Diseases. Report of a WHO Study Group.

[4] Austin G.L., Ogden L.G., Hill J.O. (2011). Trends in carbohydrate, fat, and protein intakes and association with energy intake in normal-weight, overweight, and obese individuals: 1971–2006. Am. J. Clin. Nutr..

[5] Kant A.K., Block G. (1990). Dietary vitamin B-6 intake and food sources in the US population: NHANES II, 1976–1980. Am. J. Clin. Nutr..

[6] Kit B.K., Fakhouri T.H., Park S., Nielsen S.J., Ogden C.L. (2013). Trends in sugar-sweetened beverage consumption among youth and adults in the United States: 1999–2010. Am. J. Clin. Nutr..

[7] Rehm C.D., Drewnowski A. (2016). Trends in Consumption of Solid Fats, Added Sugars, Sodium, Sugar-Sweetened Beverages, and Fruit from Fast Food Restaurants and by Fast Food Restaurant Type among US Children, 2003–2010. Nutrients.

[8] Peterkin B.B. (1986). Women’s diets: 1977 and 1985. J. Nutr. Educ..

[9] Norris J., Harnack L., Carmichael S., Pouane T., Wakimoto P., Block G. (1997). US trends in nutrient intake: The 1987 and 1992 National Health Interview Surveys. Am. J. Public Health.

[10] Centers for Disease Control and Prevention (1994). Daily dietary fat and total food-energy intakes—NHANES III, Phase 1, 1988–1991. JAMA.

[11] Funk C., Kennedy B. The New Food Fights: U.S. Public Divides over Food Science. Pew Research Center. https://www.pewresearch.org/science/2016/12/01/the-new-food-fights/.

[12] U.S. Department of Agriculture National Agricultural Library Dietary Reference Intakes. Updated 5 May 2019. https://www.nal.usda.gov/fnic/dri-nutrient-reports.

[13] Gerrior S., Juan W., Peter B. (2006). An Easy Approach to Calculating Estimated Energy Requirements. Prev. Chronic Dis..

[14] U.S. Department of Health and Human Services, U.S. Department of Agriculture (2015). 2015–2020 Dietary Guidelines for Americans.

[15] Ford E.S., Dietz W.H. (2013). Trends in energy intake among adults in the United States: Findings from NHANES. Am. J. Clin. Nutr..

[16] Layrisse M., Cook J.D., Martinez C., Roche M., Kuhn I.N., Walker R.B., Finch C.A. (1969). Food Iron Absorption: A Comparison of Vegetable and Animal Foods. Blood.

[17] Kant A.K., Graubard B.I. (2007). Secular trends in the association of socio-economic position with self-reported dietary attributes and biomarkers in the US population: National Health and Nutrition Examination Survey (NHANES) 1971–1975 to NHANES 1999–2002. Public Health Nutr..

[18] Dietz W.H., Scanlon K.S. (2012). Eliminating the Use of Partially Hydrogenated Oil in Food Production and Preparation. JAMA.

[19] National Center for Health Statistics (US) (2012). Health, United States, 2011: With Special Feature on Socioeconomic Status and Health.

[20] The Dietary Guidelines Advisory Committee (2010). Report of the Dietary Guidelines Advisory Committee on the Dietary Guidelines for Americans, 2010, to the Secretary of Agriculture and the Secretary of Health and Human Services. US Department of Agriculture Center for Nutrition Policy and Promotion Website. http://www.cnpp.usda.gov/DGAs2010-DGACReport.htm.

[21] Whelton P.K., Appel L.J., Sacco R.L., Anderson C.A.M., Antman E.M., Campbell N., Dunbar S.B., Frohlich E.D., Hall J.E., Jessup M. (2012). Sodium, Blood Pressure, and Cardiovascular Disease: Further Evidence Supporting the American Heart Association Sodium Reduction Recommendations. Circulation.

[22] Mcguire S. (2010). Strategies to Reduce Sodium Intake in the United States.

[23] Drewnowski A., Rehm C.D. (2013). Sodium Intakes of US Children and Adults from Foods and Beverages by Location of Origin and by Specific Food Source. Nutrients.

[24] Greer S., Schieb L., Schwartz G., Onufrak S., Park S. (2014). Association of the Neighborhood Retail Food Environment with Sodium and Potassium Intake among US Adults. Prev. Chronic Dis..

[25] Centers for Disease Control and Prevention (CDC) (2011). Public Health Grand Rounds: Sodium Reduction, Time for Choice.

[26] U.S. Department of Health and Human Services, General Services Administration Health and Sustainability Guidelines for Federal Concessions and Vending Operations. http://www.gsa.gov/portal/content/104429.

[27] U.S. Department of Health and Human Services, CDC Sodium Reduction in Communities. http://www.cdc.gov/dhdsp/programs/sodium_reduction.htm.

[28] U.S. Department of Health and Human Services, CDC (2010). Improving the Food Environment through Nutrition Standards: A Guide for Government Procurement.

[29] Subar A.F., Block G., James L.D. (1989). Folate intake and food sources in the US population. Am. J. Clin. Nutr..

[30] Gille D., Schmid A. (2015). Vitamin B12 in meat and dairy products. Nutr. Rev..

[31] Enstrom J.E., Kanim L.E., Klein M.A. (1992). Vitamin C Intake and Mortality among a Sample of the United States Population. Epidemiology.

[32] Booth S.L. (2012). Vitamin K: Food composition and dietary intakes. Food Nutr. Res..

[33] Frei B. (1995). Cardiovascular disease and nutrient antioxidants: Role of low-density lipoprotein oxidation. Crit. Rev. Food Sci. Nutr..

[34] Müller L., Carisveyrat C., Lowe G., Böhm V. (2016). Lycopene and its antioxidant role in the prevention of cardiovascular diseases—A critical review. Crit. Rev. Food Sci. Nutr..

[35] Hughes D.A. (2001). Dietary carotenoids and human immune function. Nutrients.

[36] Maiani G., Castón M.J., Catasta G., Toti E., Cambrodón I.G., Bysted A., Granado-Lorencio F., Olmedilla-Alonso B., Knuthsen P., Valoti M. (2010). Carotenoids: Actual knowledge on food sources, intakes, stability and bioavailability and their protective role in humans. Mol. Nutr. Food Res..

[37] Sackner-Bernstein J., Kanter D., Kaul S. (2015). Dietary Intervention for Overweight and Obese Adults: Comparison of Low-Carbohydrate and Low-Fat Diets. A Meta-Analysis. PLoS ONE.

