# Supplementary figures and images for: Trends in Dietary Nutrients by Demographic Characteristics and BMI among US Adults, 2003–2016
Source: Nutrients. 2019 Nov 1;11(11):2617. doi: 10.3390/nu11112617 (PMC6893675; doi:10.3390/nu11112617)

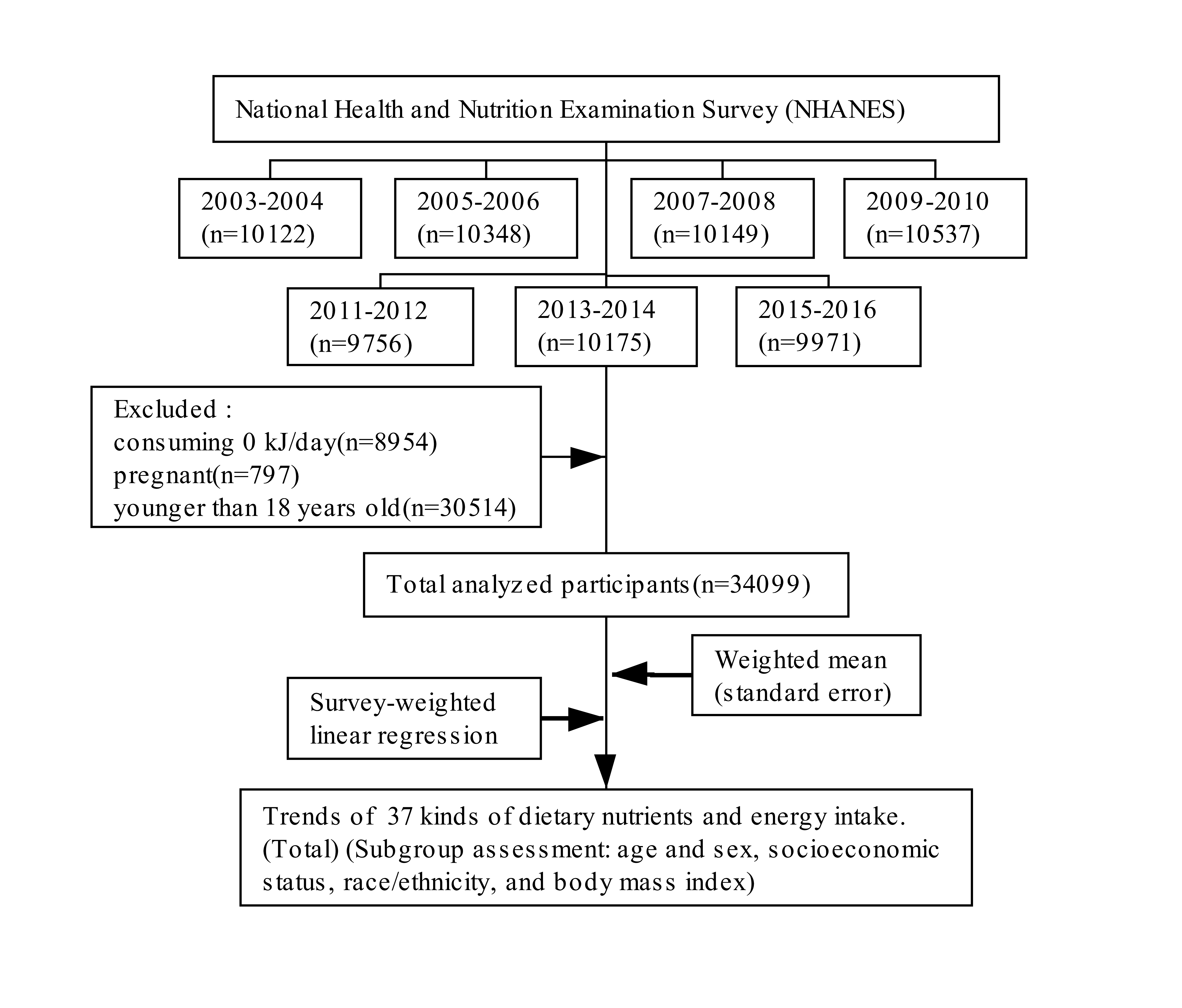

Supplement: Supplementary file 1 [file nutrients-11-02617-s001.zip › Supplementary File/Figure S1.tif]

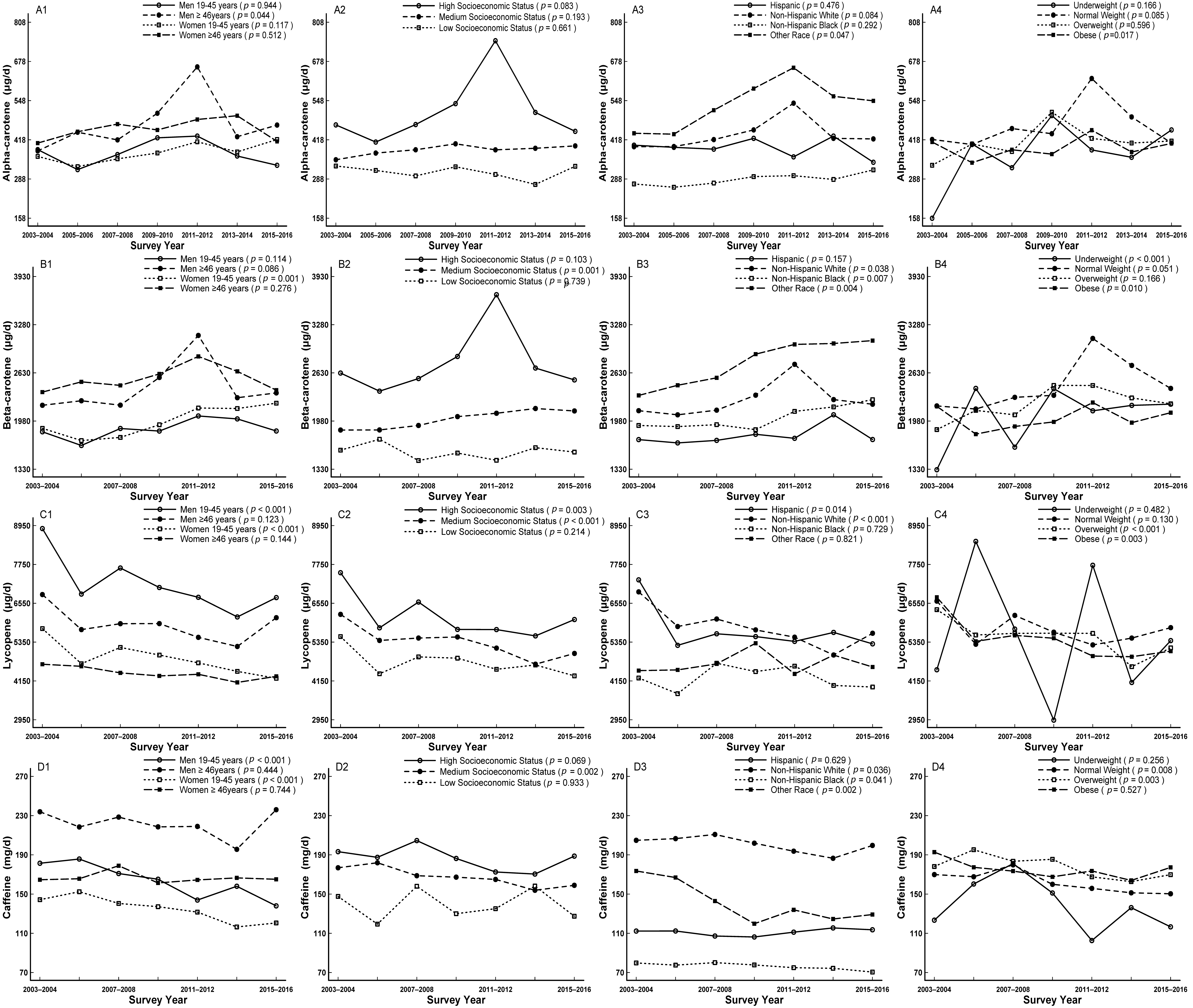

Supplement: Supplementary file 1 [file nutrients-11-02617-s001.zip › Supplementary File/FigureS2.tif]
