# Supplementary material for: Trends in Dietary Nutrients by Demographic Characteristics and BMI among US Adults, 2003–2016
Source: Nutrients. 2019 Nov 1;11(11):2617. doi: 10.3390/nu11112617 (PMC6893675; doi:10.3390/nu11112617)
Supplement: Supplementary file 1 [file nutrients-11-02617-s001.zip › Supplementary File/Supplementary Materials.doc]

Table S1 Trends in percentage of dietary nutrient intake not meeting Dietary Reference Intakes by age sex, socioeconomic status, race/ethnicity, and BMI among US adults in the National Health and Nutrition Examination Survey (NHANES 2003-2016)

|  | 2003-2004 | 2005-2006 | 2007-2008 | 2009-2010 | 2011-2012 | 2013-2014 | 2015-2016 | *P* trend | *P* value |
| --- | --- | --- | --- | --- | --- | --- | --- | --- | --- |
| **Energy (kcal/d)** |  |  |  |  |  |  |  |  |  |
| Sex Age |  |  |  |  |  |  |  |  |  |
| Men 19-45 y | 49.0% | 48.1% | 26.5% | 29.9% | 28.7% | 24.5% | 23.9% | <0.001 | <0.001 |
| Men 46+ y | 39.5% | 36.3% | 24.6% | 28.6% | 31.8% | 29.6% | 28.9% | <0.001 |  |
| Women 19-45 y | 37.0% | 33.1% | 27.2% | 26.9% | 29.9% | 29.6% | 25.3% | <0.001 |  |
| Women 46+ y | 35.6% | 33.2% | 31.7% | 35.4% | 33.9% | 34.9% | 32.6% | 0.712 |  |
| Socioeconomic status |  |  |  |  |  |  |  |  |  |
| High | 42.0% | 37.6% | 30.9% | 33.2% | 33.9% | 30.3% | 30.4% | <0.001 | <0.001 |
| Medium | 40.5% | 38.1% | 26.2% | 29.9% | 30.3% | 29.0% | 26.9% | <0.001 |  |
| Low | 32.9% | 34.6% | 25.6% | 22.2% | 26.4% | 33.6% | 26.1% | 0.198 |  |
| Race/ethnicity |  |  |  |  |  |  |  |  |  |
| Hispanic | 43.5% | 38.8% | 26.2% | 26.1% | 29.2% | 30.2% | 28.5% | <0.001 | <0.001 |
| Non-Hispanic white | 40.9% | 38.9% | 28.1% | 32.2% | 32.1% | 30.1% | 29.1% | <0.001 |  |
| Non-Hispanic black | 33.6% | 32.5% | 24.5% | 24.3% | 26.9% | 27.1% | 21.1% | <0.001 |  |
| Other race | 39.3% | 30.5% | 30.3% | 28.4% | 32.7% | 29.5% | 27.9% | 0.077 |  |
| BMI |  |  |  |  |  |  |  |  |  |
| Underweight | 61.7% | 53.2% | 52.6% | 54.4% | 58.5% | 59.8% | 57.8% | 0.806 | <0.001 |
| Normal weight | 50.4% | 49.0% | 40.2% | 39.1% | 44.1% | 40.2% | 38.6% | <0.001 |  |
| Overweight | 41.7% | 39.5% | 23.2% | 32.7% | 30.5% | 29.8% | 28.2% | <0.001 |  |
| Obese | 27.7% | 24.7% | 19.4% | 19.5% | 19.6% | 20.9% | 19.7% | <0.001 |  |
|  | **Macro-nutrients** | | | | | | | |  |
| **Protein (kcal%)** |  |  |  |  |  |  |  |  |  |
| Sex Age |  |  |  |  |  |  |  |  |  |
| Men 19-45 y | 34.6% | 32.8% | 29.4% | 30.6% | 32.4% | 29.2% | 27.4% | 0.012 | <0.001 |
| Men 46+ y | 18.6% | 20.9% | 20.4% | 19.2% | 19.8% | 17.4% | 21.3% | 0.974 |  |
| Women 19-45 y | 17.7% | 17.1% | 16.0% | 14.8% | 16.8% | 19.1% | 18.1% | 0.423 |  |
| Women 46+ y | 13.2% | 11.8% | 13.8% | 11.9% | 13.5% | 14.3% | 13.8% | 0.313 |  |
| Socioeconomic status |  |  |  |  |  |  |  |  |  |
| High | 20.1% | 18.1% | 18.4% | 17.0% | 15.6% | 15.2% | 17.0% | 0.039 | <0.001 |
| Medium | 21.2% | 22.1% | 20.1% | 20.2% | 22.6% | 21.6% | 20.8% | 0.924 |  |
| Low | 25.1% | 21.3% | 23.1% | 16.7% | 24.9% | 26.2% | 25.2% | 0.334 |  |
| Race/ethnicity |  |  |  |  |  |  |  |  |  |
| Hispanic | 20.1% | 17.8% | 19.8% | 18.0% | 20.1% | 19.7% | 19.9% | 0.640 | <0.001 |
| Non-Hispanic white | 21.2% | 20.8% | 19.2% | 18.9% | 20.7% | 19.3% | 20.1% | 0.390 |  |
| Non-Hispanic black | 25.0% | 22.6% | 26.9% | 22.3% | 24.5% | 25.3% | 20.0% | 0.086 |  |
| Other race | 15.7% | 22.1% | 13.8% | 16.1% | 12.3% | 17.1% | 17.7% | 0.848 |  |
| BMI |  |  |  |  |  |  |  |  |  |
| Underweight | 25.0% | 23.8% | 28.8% | 18.9% | 43.4% | 24.4% | 29.5% | 0.334 | <0.001 |
| Normal weight | 23.6% | 22.1% | 21.6% | 19.0% | 22.8% | 19.7% | 18.3% | 0.015 |  |
| Overweight | 20.3% | 21.1% | 17.6% | 19.8% | 19.9% | 18.6% | 18.5% | 0.279 |  |
| Obese | 19.6% | 18.8% | 20.1% | 18.2% | 18.0% | 20.8% | 21.5% | 0.222 |  |
| **Carbohydrate (kcal%)** |  |  |  |  |  |  |  |  |  |
| Sex Age |  |  |  |  |  |  |  |  |  |
| Men 19-45 y | 77.0% | 78.1% | 73.5% | 71.2% | 72.6% | 72.6% | 73.3% | 0.003 | <0.001 |
| Men 46+ y | 70.1% | 70.0% | 69.9% | 66.7% | 67.3% | 65.9% | 70.1% | 0.259 |  |
| Women 19-45 y | 59.8% | 65.3% | 61.1% | 60.6% | 62.4% | 73.2% | 62.5% | 0.571 |  |
| Women 46+ y | 66.4% | 62.0% | 65.8% | 64.5% | 59.8% | 63.3% | 59.5% | 0.026 |  |
| Socioeconomic status |  |  |  |  |  |  |  |  |  |
| High | 69.3% | 67.7% | 66.8% | 64.5% | 64.0% | 66.7% | 66.3% | 0.157 | <0.001 |
| Medium | 67.5% | 69.8% | 67.3% | 65.8% | 65.6% | 65.6% | 65.6% | 0.007 |  |
| Low | 72.0% | 65.9% | 72.1% | 69.7% | 69.8% | 68.6% | 70.1% | 0.701 |  |
| Race/ethnicity |  |  |  |  |  |  |  |  |  |
| Hispanic | 65.0% | 63.6% | 68.5% | 66.3% | 66.7% | 67.0% | 65.6% | 0.572 | 0.001 |
| Non-Hispanic white | 69.0% | 69.6% | 67.5% | 65.6% | 65.0% | 65.7% | 66.3% | 0.003 |  |
| Non-Hispanic black | 71.0% | 69.8% | 68.0% | 68.9% | 67.2% | 69.2% | 65.2% | 0.017 |  |
| Other race | 59.7% | 67.1% | 64.5% | 59.8% | 63.7% | 64.9% | 66.6% | 0.324 |  |
| BMI |  |  |  |  |  |  |  |  |  |
| Underweight | 78.1% | 69.2% | 72.7% | 65.9% | 68.3% | 58.5% | 69.3% | 0.116 | 0.027 |
| Normal weight | 64.6% | 69.4% | 65.9% | 62.4% | 63.7% | 64.0% | 63.7% | 0.066 |  |
| Overweight | 68.9% | 67.3% | 65.7% | 66.7% | 67.0% | 68.0% | 66.9% | 0.715 |  |
| Obese | 71.1% | 69.8% | 70.6% | 67.6% | 65.1% | 66.6% | 66.9% | 0.006 |  |
| **Total Fat (kcal%)** |  |  |  |  |  |  |  |  |  |
| Sex Age |  |  |  |  |  |  |  |  |  |
| Men 19-45 y | 70.0% | 70.1% | 68.9% | 69.4% | 69.7% | 69.1% | 65.6% | 0.102 | <0.001 |
| Men 46+ y | 71.4% | 64.9% | 68.9% | 66.1% | 62.8% | 65.0% | 67.1% | 0.066 |  |
| Women 19-45 y | 72.9% | 76.6% | 73.3% | 73.8% | 70.5% | 74.6% | 75.4% | 0.926 |  |
| Women 46+ y | 82.9% | 80.4% | 82.4% | 76.9% | 75.3% | 82.3% | 82.8% | 0.836 |  |
| Socioeconomic status |  |  |  |  |  |  |  |  |  |
| High | 74.6% | 69.6% | 71.5% | 67.2% | 66.7% | 69.1% | 68.1% | 0.011 | <0.001 |
| Medium | 73.6% | 74.7% | 73.7% | 73.7% | 70.5% | 74.6% | 75.2% | 0.727 |  |
| Low | 80.0% | 77.2% | 79.7% | 72.9% | 73.6% | 77.2% | 80.3% | 0.516 |  |
| Race/ethnicity |  |  |  |  |  |  |  |  |  |
| Hispanic | 74.3% | 75.4% | 72.3% | 74.3% | 70.3% | 71.9% | 72.0% | 0.147 | <0.001 |
| Non-Hispanic white | 74.4% | 72.4% | 73.4% | 70.4% | 68.2% | 72.8% | 72.5% | 0.168 |  |
| Non-Hispanic black | 75.1% | 76.3% | 76.7% | 72.5% | 75.1% | 75.1% | 78.1% | 0.461 |  |
| Other race | 73.4% | 72.6% | 71.0% | 78.7% | 71.5% | 73.6% | 74.0% | 0.845 |  |
| BMI |  |  |  |  |  |  |  |  |  |
| Underweight | 76.9% | 87.1% | 80.2% | 69.6% | 65.2% | 76.9% | 79.1% | 0.157 | 0.128 |
| Normal weight | 74.8% | 73.7% | 72.4% | 72.1% | 68.3% | 73.5% | 74.3% | 0.369 |  |
| Overweight | 74.5% | 73.1% | 72.9% | 71.9% | 70.7% | 72.1% | 70.5% | 0.068 |  |
| Obese | 73.7% | 71.9% | 75.0% | 71.3% | 69.6% | 73.3% | 74.3% | 0.953 |  |
| **Total Sugars (kcal%)** |  |  |  |  |  |  |  |  |  |
| Sex Age |  |  |  |  |  |  |  |  |  |
| Men 19-45 y | 77.9% | 75.5% | 78.7% | 75.9% | 74.1% | 78.2% | 73.6% | 0.289 | <0.001 |
| Men 46+ y | 82.1% | 82.3% | 83.0% | 80.2% | 83.2% | 80.4% | 78.3% | 0.077 |  |
| Women 19-45 y | 93.8% | 93.9% | 95.1% | 95.4% | 93.0% | 91.6% | 92.4% | 0.034 |  |
| Women 46+ y | 95.8% | 96.5% | 96.0% | 96.3% | 95.4% | 95.8% | 95.3% | 0.441 |  |
| Socioeconomic status |  |  |  |  |  |  |  |  |  |
| High | 83.3% | 83.4% | 85.9% | 84.5% | 84.7% | 85.2% | 81.4% | 0.602 | <0.001 |
| Medium | 88.9% | 88.6% | 89.3% | 88.0% | 87.4% | 87.3% | 87.0% | 0.066 |  |
| Low | 92.0% | 91.9% | 90.1% | 90.9% | 88.6% | 89.4% | 89.4% | 0.104 |  |
| Race/ethnicity |  |  |  |  |  |  |  |  |  |
| Hispanic | 89.5% | 89.7% | 88.0% | 87.7% | 84.9% | 86.1% | 84.2% | <0.001 | <0.001 |
| Non-Hispanic white | 86.7% | 86.2% | 88.1% | 86.4% | 86.2% | 86.5% | 85.0% | 0.283 |  |
| Non-Hispanic black | 90.9% | 91.3% | 90.2% | 90.8% | 88.8% | 88.4% | 88.6% | 0.059 |  |
| Other race | 86.6% | 84.6% | 85.6% | 88.1% | 90.1% | 86.6% | 85.0% | 0.956 |  |
| BMI |  |  |  |  |  |  |  |  |  |
| Underweight | 85.5% | 89.9% | 91.9% | 90.7% | 80.1% | 85.9% | 90.9% | 0.884 | 0.773 |
| Normal weight | 88.7% | 88.5% | 87.9% | 89.7% | 87.1% | 87.7% | 87.0% | 0.237 |  |
| Overweight | 87.0% | 86.0% | 89.2% | 85.9% | 87.2% | 86.0% | 84.0% | 0.101 |  |
| Obese | 86.8% | 86.5% | 87.6% | 86.2% | 86.0% | 86.7% | 85.00% | 0.326 |  |
| **Dietary fiber (g/d)** |  |  |  |  |  |  |  |  |  |
| Sex Age |  |  |  |  |  |  |  |  |  |
| Men 19-45 y | 94.3% | 92.7% | 93.1% | 92.1% | 88.1% | 89.2% | 90.1% | <0.001 | <0.001 |
| Men 46+ y | 89.9% | 89.5% | 87.8% | 85.5% | 84.8% | 86.2% | 86.8% | 0.003 |  |
| Women 19-45 y | 94.5% | 95.3% | 94.0% | 91.9% | 89.9% | 90.6% | 89.0% | <0.001 |  |
| Women 46+ y | 89.1% | 89.1% | 86.5% | 82.0% | 80.6% | 86.3% | 83.6% | 0.001 |  |
| Socioeconomic status |  |  |  |  |  |  |  |  |  |
| High | 89.4% | 89.1% | 86.5% | 82.6% | 81.3% | 84.8% | 80.9% | <0.001 | <0.001 |
| Medium | 92.9% | 93.2% | 91.8% | 90.0% | 87.6% | 89.7% | 90.4% | <0.001 |  |
| Low | 94.2% | 91.2% | 93.9% | 90.3% | 88.9% | 88.3% | 87.4% | 0.003 |  |
| Race/ethnicity |  |  |  |  |  |  |  |  |  |
| Hispanic | 87.7% | 88.4% | 88.7% | 87.0% | 83.5% | 81.1% | 83.6% | <0.001 | <0.001 |
| Non-Hispanic white | 91.7% | 91.4% | 89.8% | 87.2% | 85.0% | 89.2% | 87.2% | <0.001 |  |
| Non-Hispanic black | 96.9% | 95.9% | 95.0% | 95.2% | 92.2% | 93.0% | 93.0% | <0.001 |  |
| Other race | 95.5% | 93.6% | 91.3% | 82.2% | 85.3% | 83.5% | 85.2% | <0.001 |  |
| BMI |  |  |  |  |  |  |  |  |  |
| Underweight | 88.1% | 91.0% | 86.8% | 87.2% | 93.8% | 89.5% | 79.6% | 0.446 | <0.001 |
| Normal weight | 91.8% | 90.1% | 89.2% | 86.3% | 83.7% | 84.9% | 84.3% | <0.001 |  |
| Overweight | 91.5% | 91.6% | 90.5% | 86.5% | 84.3% | 88.6% | 87.9% | <0.001 |  |
| Obese | 93.0% | 93.3% | 91.4% | 90.3% | 88.2% | 89.8% | 88.7% | <0.001 |  |
| **Saturated fatty acids (kcal%)** |  |  |  |  |  |  |  |  |  |
| Sex Age |  |  |  |  |  |  |  |  |  |
| Men 19-45 y | 34.8% | 39.9% | 45.8% | 41.2% | 39.3% | 48.5% | 52.8% | <0.001 | <0.001 |
| Men 46+ y | 58.1% | 56.2% | 58.8% | 56.4% | 52.5% | 59.0% | 62.4% | 0.183 |  |
| Women 19-45 y | 73.7% | 78.5% | 75.5% | 75.8% | 73.3% | 76.2% | 77.3% | 0.635 |  |
| Women 46+ y | 85.0% | 85.6% | 85.0% | 81.9% | 80.5% | 84.4% | 86.7% | 0.958 |  |
| Socioeconomic status |  |  |  |  |  |  |  |  |  |
| High | 59.6% | 63.4% | 63.4% | 63.8% | 59.1% | 64.3% | 68.3% | 0.020 | <0.001 |
| Medium | 63.6% | 65.8% | 68.1% | 63.8% | 62.6% | 69.4% | 71.4% | <0.001 |  |
| Low | 69.6% | 67.1% | 67.2% | 69.0% | 67.4% | 65.7% | 74.3% | 0.402 |  |
| Race/ethnicity |  |  |  |  |  |  |  |  |  |
| Hispanic | 53.0% | 58.6% | 61.2% | 57.5% | 57.6% | 61.1% | 65.3% | 0.003 | <0.001 |
| Non-Hispanic white | 64.0% | 65.9% | 68.2% | 65.7% | 63.1% | 69.7% | 72.1% | <0.001 |  |
| Non-Hispanic black | 66.3% | 63.9% | 64.8% | 64.2% | 60.5% | 64.5% | 70.9% | 0.263 |  |
| Other race | 59.8% | 69.4% | 61.9% | 62.7% | 59.3% | 64.4% | 77.4% | 0.466 |  |
| BMI |  |  |  |  |  |  |  |  |  |
| Underweight | 61.3% | 61.9% | 67.9% | 65.1% | 62.4% | 70.4% | 57.4% | 0.994 | <0.001 |
| Normal weight | 59.8% | 64.6% | 63.8% | 63.3% | 58.6% | 64.6% | 68.0% | 0.058 |  |
| Overweight | 60.2% | 61.9% | 65.6% | 61.1% | 61.6% | 65.5% | 69.8% | 0.001 |  |
| Obese | 68.7% | 68.8% | 70.1% | 67.7% | 64.7% | 71.4% | 73.4% | 0.115 |  |
|  | **Minerals** | | | | | | | |  |
| **Calcium (mg/d)** |  |  |  |  |  |  |  |  |  |
| Sex Age |  |  |  |  |  |  |  |  |  |
| Men 19-45 y | 52.2% | 49.2% | 51.2% | 42.7% | 42.7% | 48.1% | 47.7% | 0.075 | <0.001 |
| Men 46+ y | 66.5% | 62.9% | 66.1% | 57.5% | 58.9% | 58.2% | 55.5% | <0.001 |  |
| Women 19-45 y | 76.1% | 68.7% | 66.6% | 64.5% | 65.6% | 68.3% | 66.6% | 0.001 |  |
| Women 46+ y | 86.1% | 85.2% | 86.1% | 78.6% | 80.6% | 81.3% | 84.4% | 0.028 |  |
| Socioeconomic status |  |  |  |  |  |  |  |  |  |
| High | 65.3% | 59.4% | 60.1% | 54.2% | 60.0% | 62.4% | 59.5% | 0.330 | <0.001 |
| Medium | 71.5% | 69.4% | 70.8% | 63.1% | 63.4% | 65.0% | 66.6% | <0.001 |  |
| Low | 78.8% | 77.0% | 74.2% | 72.9% | 66.2% | 69.5% | 70.5% | 0.001 |  |
| Race/ethnicity |  |  |  |  |  |  |  |  |  |
| Hispanic | 72.7% | 70.2% | 66.6% | 63.1% | 57.8% | 57.7% | 61.8% | <0.001 | <0.001 |
| Non-Hispanic white | 67.6% | 63.6% | 66.4% | 57.7% | 60.1% | 63.2% | 61.6% | 0.004 |  |
| Non-Hispanic black | 82.5% | 79.7% | 75.1% | 72.3% | 74.4% | 75.9% | 79.7% | 0.081 |  |
| Other race | 76.5% | 70.5% | 72.6% | 75.1% | 74.8% | 71.9% | 72.1% | 0.611 |  |
| BMI |  |  |  |  |  |  |  |  |  |
| Underweight | 57.4% | 65.5% | 56.3% | 65.6% | 67.3% | 53.3% | 60.7% | 0.928 | <0.001 |
| Normal weight | 68.4% | 67.7% | 66.1% | 61.1% | 60.8% | 63.3% | 63.1% | 0.002 |  |
| Overweight | 69.6% | 64.5% | 68.0% | 59.5% | 61.9% | 63.8% | 65.7% | 0.092 |  |
| Obese | 73.3% | 67.5% | 69.5% | 62.6% | 64.2% | 66.3% | 64.7% | <0.001 |  |
| **Iron (mg/d)** |  |  |  |  |  |  |  |  |  |
| Sex Age |  |  |  |  |  |  |  |  |  |
| Men 19-45 y | 4.7% | 5.5% | 6.1% | 5.7% | 6.5% | 8.5% | 10.1% | <0.001 | <0.001 |
| Men 46+ y | 6.0% | 5.5% | 6.4% | 6.9% | 4.9% | 7.5% | 10.1% | 0.001 |  |
| Women 19-45 y | 81.5% | 78.5% | 79.8% | 80.5% | 81.9% | 84.5% | 85.4% | 0.002 |  |
| Women 46+ y | 30.6% | 28.0% | 28.9% | 28.8% | 28.1% | 31.4% | 35.9% | 0.021 |  |
| Socioeconomic status |  |  |  |  |  |  |  |  |  |
| High | 27.5% | 29.5% | 26.5% | 25.9% | 26.6% | 28.1% | 29.9% | 0.639 | <0.001 |
| Medium | 31.6% | 29.5% | 31.5% | 31.6% | 31.2% | 34.8% | 36.3% | <0.001 |  |
| Low | 41.5% | 32.0% | 37.7% | 40.5% | 30.9% | 36.6% | 42.1% | 0.919 |  |
| Race/ethnicity |  |  |  |  |  |  |  |  |  |
| Hispanic | 36.9% | 34.0% | 36.3% | 37.0% | 33.2% | 36.7% | 40.2% | 0.090 | <0.001 |
| Non-Hispanic white | 28.6% | 27.0% | 27.6% | 27.5% | 26.4% | 30.2% | 31.4% | 0.054 |  |
| Non-Hispanic black | 40.3% | 37.7% | 41.1% | 39.9% | 41.4% | 40.9% | 44.8% | 0.054 |  |
| Other race | 35.5% | 40.4% | 31.3% | 35.0% | 36.3% | 36.0% | 35.4% | 0.875 |  |
| BMI |  |  |  |  |  |  |  |  |  |
| Underweight | 46.7% | 49.0% | 43.2% | 46.8% | 41.7% | 29.5% | 41.6% | 0.072 | <0.001 |
| Normal weight | 36.0% | 35.9% | 33.0% | 37.6% | 32.2% | 37.4% | 38.4% | 0.403 |  |
| Overweight | 24.9% | 23.1% | 27.3% | 24.9% | 25.9% | 27.0% | 30.1% | 0.010 |  |
| Obese | 32.0% | 29.2% | 30.8% | 29.3% | 30.5% | 34.3% | 35.1% | 0.010 |  |
| **Magnesium (mg/d)** |  |  |  |  |  |  |  |  |  |
| Sex Age |  |  |  |  |  |  |  |  |  |
| Men 19-45 y | 78.2% | 73.9% | 77.3% | 72.9% | 71.3% | 74.2% | 75.9% | 0.214 | <0.001 |
| Men 46+ y | 82.1% | 78.6% | 79.7% | 77.9% | 77.3% | 78.9% | 77.3% | 0.054 |  |
| Women 19-45 y | 80.5% | 75.9% | 75.8% | 74.7% | 72.0% | 74.1% | 71.7% | 0.007 |  |
| Women 46+ y | 83.8% | 76.8% | 75.6% | 72.7% | 70.4% | 73.2% | 72.8% | <0.001 |  |
| Socioeconomic status |  |  |  |  |  |  |  |  |  |
| High | 74.6% | 68.8% | 69.1% | 68.1% | 64.4% | 68.3% | 66.2% | 0.005 | <0.001 |
| Medium | 83.0% | 79.6% | 80.0% | 76.4% | 75.8% | 77.9% | 78.1% | 0.003 |  |
| Low | 90.6% | 82.8% | 85.4% | 83.6% | 83.9% | 81.7% | 82.1% | 0.025 |  |
| Race/ethnicity |  |  |  |  |  |  |  |  |  |
| Hispanic | 79.5% | 77.0% | 79.6% | 75.9% | 72.8% | 70.5% | 73.1% | <0.001 | <0.001 |
| Non-Hispanic white | 79.8% | 74.3% | 74.8% | 72.6% | 71.2% | 74.8% | 73.7% | 0.019 |  |
| Non-Hispanic black | 90.5% | 88.7% | 87.2% | 86.2% | 81.5% | 83.3% | 84.4% | <0.001 |  |
| Other race | 84.1% | 75.5% | 78.1% | 72.0% | 72.3% | 74.0% | 69.8% | 0.006 |  |
| BMI |  |  |  |  |  |  |  |  |  |
| Underweight | 78.3% | 76.5% | 76.5% | 65.0% | 72.6% | 70.9% | 68.3% | 0.162 | <0.001 |
| Normal weight | 79.2% | 72.9% | 71.5% | 71.8% | 67.2% | 69.1% | 68.2% | <0.001 |  |
| Overweight | 80.0% | 76.3% | 79.7% | 72.8% | 72.4% | 74.7% | 75.7% | 0.016 |  |
| Obese | 84.3% | 79.3% | 79.5% | 78.8% | 77.6% | 79.9% | 77.9% | 0.017 |  |
| **Phosphorus (mg/d)** |  |  |  |  |  |  |  |  |  |
| Sex Age |  |  |  |  |  |  |  |  |  |
| Men 19-45 y | 3.7% | 3.3% | 3.5% | 2.5% | 3.7% | 3.6% | 4.0% | 0.676 | <0.001 |
| Men 46+ y | 5.6% | 6.2% | 5.6% | 3.9% | 2.8% | 4.4% | 4.8% | 0.039 |  |
| Women 19-45 y | 11.2% | 15.7% | 13.8% | 9.2% | 12.2% | 9.8% | 12.1% | 0.105 |  |
| Women 46+ y | 18.2% | 13.1% | 13.2% | 10.5% | 11.2% | 11.9% | 12.7% | 0.013 |  |
| Socioeconomic status |  |  |  |  |  |  |  |  |  |
| High | 5.8% | 5.8% | 5.3% | 3.4% | 3.5% | 3.6% | 5.2% | 0.107 | <0.001 |
| Medium | 10.4% | 11.2% | 10.0% | 7.5% | 9.1% | 8.9% | 9.5% | 0.078 |  |
| Low | 19.8% | 14.4% | 17.1% | 12.0% | 12.9% | 13.7% | 16.8% | 0.186 |  |
| Race/ethnicity |  |  |  |  |  |  |  |  |  |
| Hispanic | 9.7% | 8.3% | 10.4% | 7.9% | 6.8% | 5.6% | 9.4% | 0.142 | <0.001 |
| Non-Hispanic white | 8.2% | 8.2% | 7.3% | 4.8% | 6.6% | 6.7% | 7.2% | 0.068 |  |
| Non-Hispanic black | 19.9% | 83.0% | 17.4% | 14.9% | 11.9% | 13.4% | 15.3% | <0.001 |  |
| Other race | 10.2% | 16.8% | 12.5% | 9.3% | 11.1% | 10.2% | 8.9% | 0.032 |  |
| BMI |  |  |  |  |  |  |  |  |  |
| Underweight | 13.2% | 10.4% | 18.4% | 5.1% | 16.9% | 3.3% | 11.9% | 0.346 | <0.001 |
| Normal weight | 10.6% | 10.6% | 9.4% | 7.0% | 6.8% | 7.4% | 8.5% | 0.002 |  |
| Overweight | 8.8% | 7.7% | 8.1% | 5.5% | 7.9% | 7.2% | 8.6% | 0.727 |  |
| Obese | 9.7% | 10.5% | 9.5% | 7.5% | 7.4% | 8.2% | 8.5% | 0.069 |  |
| **Potassium (mg/d)** |  |  |  |  |  |  |  |  |  |
| Sex Age |  |  |  |  |  |  |  |  |  |
| Men 19-45 y | 89.6% | 90.2% | 91.6% | 92.4% | 90.9% | 91.0% | 93.6% | 0.023 | <0.001 |
| Men 46+ y | 91.6% | 91.0% | 92.6% | 90.6% | 92.0% | 92.4% | 92.6% | 0.266 |  |
| Women 19-45 y | 98.1% | 98.2% | 99.4% | 98.7% | 99.4% | 98.8% | 98.6% | 0.281 |  |
| Women 46+ y | 98.2% | 99.2% | 98.8% | 97.6% | 98.1% | 99.0% | 99.4% | 0.146 |  |
| Socioeconomic status |  |  |  |  |  |  |  |  |  |
| High | 92.7% | 94.1% | 94.6% | 95.0% | 95.0% | 95.7% | 95.5% | 0.004 | 0.001 |
| Medium | 95.0% | 95.0% | 96.2% | 94.8% | 95.1% | 95.3% | 96.4% | 0.192 |  |
| Low | 96.9% | 94.1% | 96.3% | 95.8% | 96.3% | 94.9% | 97.4% | 0.735 |  |
| Race/ethnicity |  |  |  |  |  |  |  |  |  |
| Hispanic | 95.0% | 96.1% | 95.9% | 94.0% | 94.0% | 94.2% | 96.8% | 0.846 | <0.001 |
| Non-Hispanic white | 94.0% | 94.1% | 95.3% | 94.7% | 95.1% | 95.7% | 95.7% | 0.004 |  |
| Non-Hispanic black | 97.1% | 97.0% | 98.0% | 97.3% | 96.5% | 95.7% | 97.8% | 0.495 |  |
| Other race | 93.6% | 95.0% | 95.9% | 94.1% | 95.7% | 94.2% | 96.3% | 0.427 |  |
| BMI |  |  |  |  |  |  |  |  |  |
| Underweight | 92.3% | 91.2% | 99.3% | 94.4% | 98.4% | 90.1% | 97.6% | 0.473 | <0.001 |
| Normal weight | 94.5% | 94.4% | 94.3% | 94.9% | 94.8% | 94.9% | 95.1% | 0.451 |  |
| Overweight | 93.2% | 94.1% | 96.1% | 94.5% | 94.5% | 94.7% | 96.3% | 0.032 |  |
| Obese | 95.9% | 95.6% | 96.3% | 95.3% | 95.9% | 96.6% | 96.7% | 0.205 |  |
| **Sodium (mg/d)** |  |  |  |  |  |  |  |  |  |
| Sex Age |  |  |  |  |  |  |  |  |  |
| Men 19-45 y | 90.9% | 89.5% | 89.2% | 91.9% | 91.3% | 92.5% | 90.9% | 0.205 | <0.001 |
| Men 46+ y | 81.6% | 84.7% | 83.7% | 88.2% | 88.2% | 88.3% | 86.5% | 0.001 |  |
| Women 19-45 y | 74.3% | 71.1% | 72.4% | 76.1% | 75.3% | 75.2% | 73.8% | 0.298 |  |
| Women 46+ y | 61.5% | 63.6% | 62.5% | 69.7% | 69.6% | 67.2% | 66.6% | 0.007 |  |
| Socioeconomic status |  |  |  |  |  |  |  |  |  |
| High | 84.0% | 82.6% | 81.6% | 86.8% | 84.0% | 85.1% | 85.1% | 0.132 | <0.001 |
| Medium | 75.3% | 75.5% | 75.7% | 79.6% | 80.5% | 78.9% | 76.7% | 0.021 |  |
| Low | 63.9% | 64.3% | 66.1% | 72.0% | 69.7% | 72.9% | 67.1% | 0.018 |  |
| Race/ethnicity |  |  |  |  |  |  |  |  |  |
| Hispanic | 74.1% | 72.0% | 70.8% | 75.3% | 79.8% | 78.2% | 75.9% | 0.006 | <0.001 |
| Non-Hispanic white | 78.3% | 78.9% | 78.0% | 83.2% | 81.6% | 82.2% | 79.9% | 0.015 |  |
| Non-Hispanic black | 69.0% | 71.5% | 71.2% | 74.2% | 77.7% | 73.7% | 73.5% | 0.013 |  |
| Other race | 82.0% | 75.1% | 84.5% | 83.9% | 80.3% | 78.9% | 82.8% | 0.706 |  |
| BMI |  |  |  |  |  |  |  |  |  |
| Underweight | 63.9% | 68.7% | 70.9% | 73.7% | 80.7% | 81.1% | 78.9% | 0.004 | 0.023 |
| Normal weight | 76.2% | 75.5% | 77.1% | 79.1% | 80.4% | 80.5% | 80.1% | 0.002 |  |
| Overweight | 77.2% | 77.9% | 77.0% | 83.1% | 80.4% | 80.6% | 76.9% | 0.349 |  |
| Obese | 78.4% | 78.3% | 76.4% | 81.6% | 81.5% | 80.3% | 79.7% | 0.124 |  |
| **Zinc (mg/d)** |  |  |  |  |  |  |  |  |  |
| Sex Age |  |  |  |  |  |  |  |  |  |
| Men 19-45 y | 28.6% | 31.4% | 33.8% | 32.8% | 36.7% | 40.2% | 39.5% | <0.001 | <0.001 |
| Men 46+ y | 40.9% | 35.5% | 43.0% | 39.3% | 41.5% | 44.2% | 43.7% | 0.048 |  |
| Women 19-45 y | 33.5% | 38.8% | 37.7% | 37.6% | 41.0% | 40.0% | 37.9% | 0.072 |  |
| Women 46+ y | 40.3% | 39.7% | 38.3% | 37.7% | 40.4% | 41.3% | 43.9% | 0.129 |  |
| Socioeconomic status |  |  |  |  |  |  |  |  |  |
| High | 28.7% | 31.0% | 32.8% | 32.8% | 32.6% | 37.2% | 32.9% | 0.018 | <0.001 |
| Medium | 36.9% | 38.3% | 39.8% | 37.2% | 43.0% | 42.9% | 44.8% | <0.001 |  |
| Low | 53.4% | 45.5% | 45.5% | 49.7% | 47.3% | 48.4% | 54.3% | 0.640 |  |
| Race/ethnicity |  |  |  |  |  |  |  |  |  |
| Hispanic | 36.9% | 39.7% | 41.6% | 42.9% | 39.1% | 36.8% | 42.9% | 0.403 | <0.001 |
| Non-Hispanic white | 33.3% | 34.1% | 35.0% | 33.1% | 38.0% | 40.0% | 38.8% | 0.001 |  |
| Non-Hispanic black | 47.2% | 45.4% | 48.6% | 48.7% | 47.7% | 51.5% | 56.2% | 0.002 |  |
| Other race | 41.7% | 41.6% | 48.1% | 44.3% | 47.3% | 48.4% | 40.5% | 0.892 |  |
| BMI |  |  |  |  |  |  |  |  |  |
| Underweight | 28.6% | 31.1% | 37.2% | 34.2% | 39.4% | 35.8% | 33.5% | 0.410 | <0.001 |
| Normal weight | 36.8% | 36.6% | 34.2% | 38.0% | 39.1% | 39.5% | 38.3% | 0.126 |  |
| Overweight | 37.3% | 37.8% | 41.8% | 35.8% | 42.0% | 42.2% | 42.8% | 0.025 |  |
| Obese | 33.6% | 34.9% | 38.0% | 37.1% | 38.6% | 42.4% | 42.9% | <0.001 |  |
| **Copper (mg/d)** |  |  |  |  |  |  |  |  |  |
| Sex Age |  |  |  |  |  |  |  |  |  |
| Men 19-45 y | 16.1% | 14.4% | 15.8% | 17.7% | 20.6% | 25.7% | 23.6% | <0.001 | <0.001 |
| Men 46+ y | 20.8% | 13.1% | 17.6% | 16.1% | 20.6% | 20.9% | 23.6% | 0.001 |  |
| Women 19-45 y | 36.8% | 34.8% | 34.7% | 35.9% | 37.8% | 41.3% | 37.6% | 0.094 |  |
| Women 46+ y | 40.7% | 27.8% | 31.6% | 29.5% | 34.4% | 35.4% | 37.3% | 0.375 |  |
| Socioeconomic status |  |  |  |  |  |  |  |  |  |
| High | 21.3% | 14.6% | 15.8% | 14.0% | 18.0% | 21.6% | 18.6% | 0.266 | <0.001 |
| Medium | 30.3% | 25.6% | 27.8% | 27.9% | 32.4% | 34.3% | 35.4% | <0.001 |  |
| Low | 46.0% | 36.5% | 39.6% | 43.4% | 41.7% | 44.6% | 49.0% | 0.073 |  |
| Race/ethnicity |  |  |  |  |  |  |  |  |  |
| Hispanic | 28.3% | 23.6% | 27.4% | 29.9% | 29.4% | 30.2% | 33.9% | 0.002 | <0.001 |
| Non-Hispanic white | 26.4% | 20.5% | 23.2% | 22.3% | 26.8% | 29.5% | 27.9% | 0.002 |  |
| Non-Hispanic black | 46.4% | 33.4% | 36.3% | 39.9% | 37.6% | 43.8% | 48.2% | 0.055 |  |
| Other race | 27.4% | 27.2% | 20.2% | 17.2% | 27.3% | 26.6% | 24.6% | 0.882 |  |
| BMI |  |  |  |  |  |  |  |  |  |
| Underweight | 38.3% | 27.4% | 31.2% | 29.6% | 30.7% | 32.2% | 27.2% | 0.482 | <0.001 |
| Normal weight | 27.5% | 24.0% | 22.9% | 26.6% | 24.0% | 28.5% | 26.8% | 0.413 |  |
| Overweight | 27.0% | 21.1% | 25.3% | 21.2% | 28.0% | 29.5% | 31.1% | 0.002 |  |
| Obese | 31.4% | 22.6% | 26.6% | 26.7% | 32.4% | 33.8% | 33.1% | <0.001 |  |
| **Selenium (µg/d)** |  |  |  |  |  |  |  |  |  |
| Sex Age |  |  |  |  |  |  |  |  |  |
| Men 19-45 y | 3.1% | 4.4% | 3.4% | 3.6% | 3.4% | 4.0% | 3.9% | 0.639 | <0.001 |
| Men 46+ y | 5.8% | 6.1% | 6.0% | 4.4% | 3.3% | 4.5% | 4.2% | 0.007 |  |
| Women 19-45 y | 11.7% | 16.2% | 14.8% | 10.2% | 12.8% | 10.9% | 11.5% | 0.049 |  |
| Women 46+ y | 18.2% | 13.1% | 16.7% | 14.1% | 13.2% | 12.8% | 12.6% | 0.006 |  |
| Socioeconomic status |  |  |  |  |  |  |  |  |  |
| High | 7.3% | 6.3% | 7.2% | 6.3% | 5.2% | 5.2% | 5.8% | 0.052 | <0.001 |
| Medium | 9.9% | 11.6% | 11.2% | 8.8% | 9.4% | 9.2% | 8.9% | 0.012 |  |
| Low | 18.9% | 14.0% | 15.7% | 11.3% | 12.8% | 13.3% | 15.0% | 0.140 |  |
| Race/ethnicity |  |  |  |  |  |  |  |  |  |
| Hispanic | 7.5% | 10.5% | 9.8% | 8.3% | 7.6% | 7.0% | 8.4% | 0.181 | <0.001 |
| Non-Hispanic white | 9.8% | 9.4% | 10.4% | 7.6% | 8.3% | 7.9% | 7.6% | 0.003 |  |
| Non-Hispanic black | 14.6% | 12.9% | 14.1% | 12.3% | 9.0% | 12.7% | 13.4% | 0.222 |  |
| Other race | 4.3% | 11.1% | 3.8% | 7.0% | 8.4% | 6.2% | 5.8% | 0.796 |  |
| BMI |  |  |  |  |  |  |  |  |  |
| Underweight | 12.8% | 11.6% | 17.4% | 5.9% | 11.9% | 4.0% | 9.8% | 0.125 | 0.350 |
| Normal weight | 10.0% | 11.2% | 10.7% | 9.3% | 8.1% | 9.0% | 7.7% | 0.005 |  |
| Overweight | 9.7% | 9.2% | 10.0% | 6.9% | 9.2% | 7.3% | 8.5% | 0.090 |  |
| Obese | 9.5% | 9.5% | 10.1% | 8.7% | 7.4% | 8.5% | 8.3% | 0.101 |  |
|  | **Vitamins** | | | | | | | |  |
| **Vitamin A (µg/d)** |  |  |  |  |  |  |  |  |  |
| Sex Age |  |  |  |  |  |  |  |  |  |
| Men 19-45 y | 78.9% | 77.1% | 78.7% | 78.8% | 79.2% | 75.9% | 78.5% | 0.676 | <0.001 |
| Men 46+ y | 79.0% | 75.6% | 73.6% | 75.0% | 73.2% | 76.3% | 76.0% | 0.517 |  |
| Women 19-45 y | 75.7% | 75.1% | 73.1% | 72.8% | 71.7% | 73.9% | 73.0% | 0.190 |  |
| Women 46+ y | 69.4% | 65.8% | 65.3% | 65.6% | 67.1% | 65.5% | 72.2% | 0.253 |  |
| Socioeconomic status |  |  |  |  |  |  |  |  |  |
| High | 68.9% | 69.4% | 65.1% | 66.4% | 67.0% | 68.2% | 70.8% | 0.545 | <0.001 |
| Medium | 77.7% | 74.7% | 74.5% | 74.6% | 74.4% | 73.9% | 76.1% | 0.338 |  |
| Low | 85.4% | 83.6% | 85.9% | 83.8% | 82.7% | 82.5% | 83.7% | 0.270 |  |
| Race/ethnicity |  |  |  |  |  |  |  |  |  |
| Hispanic | 83.9% | 83.4% | 81.6% | 82.4% | 81.9% | 77.1% | 82.0% | 0.046 | <0.001 |
| Non-Hispanic white | 73.1% | 70.8% | 69.1% | 69.4% | 69.3% | 70.3% | 72.4% | 0.741 |  |
| Non-Hispanic black | 81.3% | 81.3% | 80.8% | 80.1% | 80.0% | 79.8% | 80.2% | 0.315 |  |
| Other race | 83.6% | 73.0% | 78.5% | 78.6% | 75.9% | 75.5% | 74.1% | 0.070 |  |
| BMI |  |  |  |  |  |  |  |  |  |
| Underweight | 80.7% | 71.4% | 69.7% | 68.5% | 72.3% | 67.8% | 56.8% | 0.026 | <0.001 |
| Normal weight | 73.7% | 69.8% | 68.8% | 71.5% | 70.2% | 67.1% | 71.5% | 0.260 |  |
| Overweight | 75.1% | 74.6% | 72.7% | 71.8% | 72.2% | 74.1% | 74.7% | 0.795 |  |
| Obese | 78.1% | 75.9% | 76.0% | 75.3% | 75.0% | 75.7% | 77.8% | 0.925 |  |
| **Vitamin B_1_ (mg/d)** |  |  |  |  |  |  |  |  |  |
| Sex Age |  |  |  |  |  |  |  |  |  |
| Men 19-45 y | 15.6% | 15.8% | 19.4% | 16.9% | 19.3% | 18.8% | 21.5% | 0.001 | <0.001 |
| Men 46+ y | 20.3% | 20.1% | 20.8% | 18.7% | 18.2% | 22.0% | 20.9% | 0.673 |  |
| Women 19-45 y | 33.3% | 34.8% | 34.7% | 31.0% | 35.3% | 34.8% | 35.0% | 0.592 |  |
| Women 46+ y | 35.5% | 35.4% | 33.4% | 30.8% | 34.6% | 34.9% | 39.6% | 0.172 |  |
| Socioeconomic status |  |  |  |  |  |  |  |  |  |
| High | 21.0% | 22.2% | 20.4% | 19.4% | 24.1% | 24.3% | 23.4% | 0.097 | <0.001 |
| Medium | 27.3% | 27.7% | 29.1% | 25.5% | 27.4% | 29.1% | 31.9% | 0.015 |  |
| Low | 37.8% | 39.7% | 38.9% | 36.1% | 36.2% | 33.5% | 40.9% | 0.608 |  |
| Race/ethnicity |  |  |  |  |  |  |  |  |  |
| Hispanic | 26.2% | 29.5% | 30.1% | 29.6% | 28.0% | 26.0% | 31.1% | 0.559 | <0.001 |
| Non-Hispanic white | 23.9% | 24.2% | 24.7% | 21.8% | 25.3% | 26.8% | 27.9% | 0.010 |  |
| Non-Hispanic black | 41.1% | 39.1% | 41.1% | 35.6% | 35.3% | 37.1% | 41.0% | 0.429 |  |
| Other race | 28.5% | 27.8% | 23.7% | 24.2% | 28.1% | 27.5% | 26.3% | 0.956 |  |
| BMI |  |  |  |  |  |  |  |  |  |
| Underweight | 33.9% | 25.3% | 31.5% | 36.1% | 29.7% | 17.0% | 23.1% | 0.078 | <0.001 |
| Normal weight | 24.8% | 27.5% | 26.2% | 24.8% | 26.7% | 27.1% | 27.8% | 0.261 |  |
| Overweight | 26.3% | 24.0% | 27.2% | 21.5% | 27.0% | 26.9% | 28.2% | 0.201 |  |
| Obese | 27.3% | 28.4% | 27.9% | 26.5% | 27.2% | 29.6% | 32.2% | 0.068 |  |
| **Vitamin B_2_ (mg/d)** |  |  |  |  |  |  |  |  |  |
| Sex Age |  |  |  |  |  |  |  |  |  |
| Men 19-45 y | 9.7% | 11.0% | 12.3% | 14.1% | 15.6% | 14.2% | 15.5% | <0.001 | <0.001 |
| Men 46+ y | 7.9% | 9.2% | 11.0% | 11.0% | 9.4% | 10.7% | 9.7% | 0.616 |  |
| Women 19-45 y | 15.0% | 17.0% | 17.3% | 17.1% | 20.3% | 16.7% | 17.9% | 0.129 |  |
| Women 46+ y | 14.9% | 13.2% | 12.1% | 14.4% | 13.6% | 13.4% | 14.8% | 0.771 |  |
| Socioeconomic status |  |  |  |  |  |  |  |  |  |
| High | 7.6% | 8.6% | 7.3% | 8.7% | 9.6% | 8.3% | 8.2% | 0.502 | <0.001 |
| Medium | 12.5% | 13.7% | 14.5% | 15.3% | 16.4% | 15.7% | 16.3% | 0.006 |  |
| Low | 25.0% | 23.7% | 25.6% | 26.0% | 22.3% | 21.9% | 28.0% | 0.990 |  |
| Race/ethnicity |  |  |  |  |  |  |  |  |  |
| Hispanic | 15.8% | 19.6% | 19.5% | 21.0% | 16.1% | 14.5% | 19.8% | 0.754 | <0.001 |
| Non-Hispanic white | 8.8% | 9.2% | 9.4% | 9.6% | 11.1% | 10.8% | 10.2% | 0.037 |  |
| Non-Hispanic black | 25.8% | 24.9% | 27.3% | 28.6% | 28.6% | 26.8% | 28.6% | 0.185 |  |
| Other race | 19.3% | 19.6% | 17.4% | 24.3% | 22.9% | 19.6% | 18.5% | 0.962 |  |
| BMI |  |  |  |  |  |  |  |  |  |
| Underweight | 15.4% | 18.0% | 17.0% | 17.1% | 17.3% | 7.8% | 14.7% | 0.262 | <0.001 |
| Normal weight | 10.6% | 12.9% | 13.1% | 14.6% | 13.7% | 13.6% | 12.9% | 0.204 |  |
| Overweight | 12.5% | 11.7% | 13.2% | 12.7% | 14.4% | 13.6% | 14.4% | 0.162 |  |
| Obese | 12.5% | 13.1% | 13.2% | 15.0% | 15.5% | 14.2% | 15.3% | 0.052 |  |
| **Vitamin B_3_ (mg/d)** |  |  |  |  |  |  |  |  |  |
| Sex Age |  |  |  |  |  |  |  |  |  |
| Men 19-45 y | 8.6% | 7.6% | 8.5% | 7.0% | 8.5% | 9.1% | 10.1% | 0.137 | <0.001 |
| Men 46+ y | 13.4% | 11.3% | 13.7% | 11.3% | 9.1% | 9.0% | 11.7% | 0.030 |  |
| Women 19-45 y | 20.3% | 21.6% | 20.3% | 15.7% | 19.5% | 18.4% | 18.8% | 0.210 |  |
| Women 46+ y | 26.4% | 22.4% | 23.9% | 20.3% | 19.8% | 19.2% | 23.8% | 0.080 |  |
| Socioeconomic status |  |  |  |  |  |  |  |  |  |
| High | 12.7% | 11.3% | 11.3% | 9.5% | 10.1% | 9.1% | 11.9% | 0.267 | <0.001 |
| Medium | 17.7% | 17.3% | 18.0% | 14.6% | 15.6% | 15.8% | 18.0% | 0.398 |  |
| Low | 30.7% | 24.6% | 27.9% | 22.6% | 22.6% | 22.2% | 25.2% | 0.057 |  |
| Race/ethnicity |  |  |  |  |  |  |  |  |  |
| Hispanic | 18.4% | 20.2% | 20.0% | 16.7% | 15.4% | 14.3% | 17.3% | 0.016 | <0.001 |
| Non-Hispanic white | 16.0% | 14.3% | 15.1% | 11.6% | 13.4% | 13.1% | 15.9% | 0.531 |  |
| Non-Hispanic black | 24.2% | 19.9% | 23.3% | 21.5% | 15.8% | 18.4% | 20.9% | 0.006 |  |
| Other race | 17.6% | 18.1% | 16.0% | 17.0% | 18.8% | 15.7% | 13.6% | 0.272 |  |
| BMI |  |  |  |  |  |  |  |  |  |
| Underweight | 18.0% | 17.8% | 23.0% | 15.4% | 14.5% | 6.9% | 14.0% | 0.029 | <0.001 |
| Normal weight | 17.7% | 15.7% | 15.7% | 13.8% | 14.9% | 13.8% | 16.0% | 0.176 |  |
| Overweight | 16.5% | 15.0% | 16.5% | 12.9% | 13.8% | 12.9% | 16.6% | 0.311 |  |
| Obese | 17.5% | 16.4% | 17.7% | 14.3% | 14.3% | 15.5% | 16.7% | 0.421 |  |
| **Vitamin B_6_ (mg/d)** |  |  |  |  |  |  |  |  |  |
| Sex Age |  |  |  |  |  |  |  |  |  |
| Men 19-45 y | 15.9% | 13.2% | 14.4% | 12.2% | 12.1% | 13.4% | 15.8% | 0.760 | <0.001 |
| Men 46+ y | 33.4% | 27.6% | 29.3% | 27.6% | 26.2% | 24.8% | 28.6% | 0.029 |  |
| Women 19-45 y | 40.1% | 39.5% | 34.0% | 31.8% | 34.7% | 30.3% | 30.9% | <0.001 |  |
| Women 46+ y | 47.1% | 42.6% | 44.4% | 39.5% | 36.8% | 39.3% | 44.0% | 0.075 |  |
| Socioeconomic status |  |  |  |  |  |  |  |  |  |
| High | 28.5% | 25.2% | 24.6% | 23.3% | 21.5% | 20.2% | 24.3% | 0.012 | <0.001 |
| Medium | 35.1% | 32.8% | 32.2% | 29.0% | 29.9% | 29.4% | 32.9% | 0.039 |  |
| Low | 46.6% | 39.3% | 42.2% | 37.9% | 36.2% | 41.6% | 41.2% | 0.174 |  |
| Race/ethnicity |  |  |  |  |  |  |  |  |  |
| Hispanic | 30.1% | 30.6% | 28.3% | 26.0% | 22.2% | 24.0% | 29.3% | 0.114 | <0.001 |
| Non-Hispanic white | 33.2% | 29.6% | 30.1% | 26.9% | 28.0% | 27.4% | 30.0% | 0.085 |  |
| Non-Hispanic black | 40.9% | 35.9% | 37.9% | 34.5% | 31.3% | 32.3% | 39.8% | 0.130 |  |
| Other race | 39.4% | 35.3% | 28.9% | 32.8% | 28.3% | 25.6% | 25.4% | <0.001 |  |
| BMI |  |  |  |  |  |  |  |  |  |
| Underweight | 35.4% | 33.6% | 44.6% | 32.2% | 25.5% | 27.4% | 32.2% | 0.249 | <0.001 |
| Normal weight | 32.9% | 30.7% | 28.6% | 27.0% | 27.7% | 24.4% | 27.4% | 0.004 |  |
| Overweight | 34.0% | 29.0% | 29.6% | 26.8% | 25.4% | 26.9% | 28.8% | 0.015 |  |
| Obese | 35.1% | 32.1% | 33.1% | 29.7% | 29.8% | 29.7% | 34.1% | 0.268 |  |
| **Vitamin B_12_ (µg/d)** |  |  |  |  |  |  |  |  |  |
| Sex Age |  |  |  |  |  |  |  |  |  |
| Men 19-45 y | 12.8% | 12.4% | 11.4% | 10.9% | 12.3% | 13.8% | 18.1% | 0.003 | <0.001 |
| Men 46+ y | 14.5% | 13.3% | 13.6% | 11.7% | 12.9% | 16.9% | 16.0% | 0.112 |  |
| Women 19-45 y | 26.8% | 28.0% | 26.1% | 23.1% | 30.1% | 29.4% | 28.6% | 0.133 |  |
| Women 46+ y | 27.9% | 24.6% | 24.7% | 23.4% | 24.6% | 29.4% | 32.0% | 0.016 |  |
| Socioeconomic status |  |  |  |  |  |  |  |  |  |
| High | 18.1% | 15.4% | 17.6% | 14.8% | 16.5% | 19.7% | 19.3% | 0.054 | 0.001 |
| Medium | 20.5% | 21.2% | 18.8% | 17.7% | 21.7% | 23.4% | 25.7% | <0.001 |  |
| Low | 31.2% | 27.3% | 26.9% | 25.5% | 21.9% | 28.0% | 31.8% | 0.818 |  |
| Race/ethnicity |  |  |  |  |  |  |  |  |  |
| Hispanic | 22.6% | 25.2% | 23.0% | 22.6% | 19.4% | 23.3% | 26.2% | 0.549 | <0.001 |
| Non-Hispanic white | 18.9% | 17.3% | 16.8% | 14.4% | 18.8% | 21.2% | 21.7% | 0.002 |  |
| Non-Hispanic black | 28.6% | 26.8% | 26.5% | 27.4% | 25.3% | 27.7% | 31.9% | 0.216 |  |
| Other race | 24.5% | 26.0% | 23.5% | 22.1% | 23.9% | 25.6% | 27.2% | 0.557 |  |
| BMI |  |  |  |  |  |  |  |  |  |
| Underweight | 22.3% | 18.4% | 29.6% | 18.3% | 19.1% | 18.3% | 26.9% | 0.903 | 0.267 |
| Normal weight | 22.8% | 20.6% | 18.8% | 19.9% | 21.5% | 24.7% | 21.8% | 0.273 |  |
| Overweight | 19.8% | 18.4% | 18.5% | 15.7% | 20.6% | 21.7% | 23.2% | 0.035 |  |
| Obese | 19.2% | 20.0% | 19.4% | 17.0% | 18.2% | 21.9% | 25.9% | <0.001 |  |
| **Folate DFE (µg/d)** |  |  |  |  |  |  |  |  |  |
| Sex Age |  |  |  |  |  |  |  |  |  |
| Men 19-45 y | 22.4% | 23.0% | 26.6% | 22.2% | 22.7% | 29.5% | 29.5% | 0.001 | <0.001 |
| Men 46+ y | 30.9% | 28.9% | 30.3% | 28.0% | 26.7% | 32.9% | 32.7% | 0.300 |  |
| Women 19-45 y | 46.5% | 47.1% | 42.9% | 42.0% | 44.7% | 46.8% | 48.9% | 0.451 |  |
| Women 46+ y | 47.9% | 48.3% | 48.2% | 46.9% | 46.4% | 49.0% | 55.1% | 0.031 |  |
| Socioeconomic status |  |  |  |  |  |  |  |  |  |
| High | 30.8% | 30.7% | 29.8% | 26.4% | 29.0% | 35.9% | 34.0% | 0.042 | <0.001 |
| Medium | 38.2% | 39.0% | 39.3% | 37.2% | 37.7% | 41.2% | 45.6% | <0.001 |  |
| Low | 51.0% | 47.6% | 48.7% | 51.2% | 44.0% | 45.7% | 50.8% | 0.496 |  |
| Race/ethnicity |  |  |  |  |  |  |  |  |  |
| Hispanic | 35.4% | 40.3% | 39.5% | 35.8% | 33.4% | 36.3% | 39.0% | 0.798 | <0.001 |
| Non-Hispanic white | 35.3% | 34.9% | 35.1% | 33.1% | 33.6% | 39.7% | 42.1% | 0.003 |  |
| Non-Hispanic black | 51.4% | 48.3% | 49.6% | 47.8% | 48.8% | 49.3% | 53.5% | 0.457 |  |
| Other race | 33.8% | 30.6% | 31.8% | 31.7% | 34.7% | 33.7% | 32.7% | 0.719 |  |
| BMI |  |  |  |  |  |  |  |  |  |
| Underweight | 37.3% | 39.1% | 40.3% | 40.9% | 36.5% | 27.6% | 24.5% | 0.037 | <0.001 |
| Normal weight | 34.9% | 37.2% | 34.4% | 34.6% | 33.4% | 37.3% | 38.1% | 0.308 |  |
| Overweight | 34.4% | 33.1% | 36.9% | 32.7% | 34.1% | 38.7% | 41.6% | 0.008 |  |
| Obese | 41.8% | 39.7% | 39.7% | 37.2% | 38.2% | 43.1% | 45.8% | 0.020 |  |
| **Vitamin C (mg/d)** |  |  |  |  |  |  |  |  |  |
| Sex Age |  |  |  |  |  |  |  |  |  |
| Men 19-45 y | 61.7% | 61.2% | 58.9% | 63.0% | 64.6% | 67.2% | 66.6% | 0.002 | <0.001 |
| Men 46+ y | 60.6% | 58.2% | 62.7% | 60.2% | 62.9% | 64.5% | 64.5% | 0.046 |  |
| Women 19-45 y | 59.2% | 62.5% | 63.9% | 59.3% | 63.1% | 61.0% | 62.8% | 0.551 |  |
| Women 46+ y | 54.1% | 55.0% | 55.5% | 53.8% | 56.1% | 58.2% | 62.7% | <0.001 |  |
| Socioeconomic status |  |  |  |  |  |  |  |  |  |
| High | 53.8% | 54.4% | 55.5% | 54.0% | 55.0% | 57.2% | 58.3% | 0.065 | <0.001 |
| Medium | 60.5% | 61.8% | 62.1% | 60.7% | 64.6% | 64.5% | 66.8% | 0.001 |  |
| Low | 65.2% | 61.0% | 62.9% | 64.8% | 64.6% | 70.8% | 67.9% | 0.092 |  |
| Race/ethnicity |  |  |  |  |  |  |  |  |  |
| Hispanic | 48.7% | 55.9% | 55.8% | 54.7% | 55.0% | 57.9% | 60.2% | <0.001 | <0.001 |
| Non-Hispanic white | 61.5% | 60.5% | 62.5% | 59.9% | 63.7% | 64.5% | 65.9% | 0.014 |  |
| Non-Hispanic black | 52.7% | 55.6% | 52.3% | 59.2% | 57.0% | 60.9% | 63.7% | <0.001 |  |
| Other race | 57.0% | 57.5% | 55.9% | 57.5% | 59.0% | 57.2% | 56.9% | 0.933 |  |
| BMI |  |  |  |  |  |  |  |  |  |
| Underweight | 63.2% | 50.2% | 77.1% | 60.8% | 62.0% | 55.2% | 58.7% | 0.642 | <0.001 |
| Normal weight | 57.4% | 55.7% | 56.7% | 58.8% | 58.1% | 55.5% | 58.2% | 0.745 |  |
| Overweight | 56.7% | 60.0% | 60.8% | 57.8% | 61.3% | 63.4% | 63.0% | 0.017 |  |
| Obese | 62.4% | 62.3% | 61.8% | 60.1% | 64.3% | 67.5% | 69.0% | <0.001 |  |
| **Vitamin D (µg/d)** |  |  |  |  |  |  |  |  |  |
| Sex Age |  |  |  |  |  |  |  |  |  |
| Men 19-45 y | - | - | 95.7% | 95.1% | 95.8% | 94.1% | 95.1% | 0.369 | <0.001 |
| Men 46+ y | - | - | 96.2% | 94.6% | 96.1% | 94.2% | 96.8% | 0.694 |  |
| Women 19-45 y | - | - | 98.7% | 97.3% | 99.0% | 98.0% | 97.9% | 0.616 |  |
| Women 46+ y | - | - | 99.3% | 97.5% | 97.8% | 97.2% | 97.8% | 0.036 |  |
| Socioeconomic status |  |  |  |  |  |  |  |  |  |
| High | - | - | 96.8% | 94.6% | 96.9% | 94.6% | 95.8% | 0.478 | 0.001 |
| Medium | - | - | 97.7% | 96.6% | 97.2% | 96.4% | 97.7% | 0.863 |  |
| Low | - | - | 98.8% | 98.5% | 97.9% | 97.8% | 95.7% | 0.015 |  |
| Race/ethnicity |  |  |  |  |  |  |  |  |  |
| Hispanic | - | - | 97.3% | 96.7% | 96.6% | 96.0% | 97.0% | 0.571 | <0.001 |
| Non-Hispanic white | - | - | 97.3% | 95.6% | 97.1% | 95.6% | 97.0% | 0.724 |  |
| Non-Hispanic black | - | - | 98.7% | 97.9% | 98.1% | 97.4% | 97.3% | 0.025 |  |
| Other race | - | - | 98.1% | 97.6% | 97.2% | 96.3% | 95.7% | 0.049 |  |
| BMI |  |  |  |  |  |  |  |  |  |
| Underweight | - | - | 99.8% | 87.2% | 99.0% | 95.9% | 98.3% | 0.257 | <0.001 |
| Normal weight | - | - | 97.4% | 95.9% | 97.4% | 95.6% | 96.4% | 0.222 |  |
| Overweight | - | - | 96.8% | 95.7% | 96.8% | 95.1% | 97.0% | 0.885 |  |
| Obese | - | - | 98.3% | 97.3% | 97.2% | 96.9% | 97.2% | 0.166 |  |
| **Vitamin E (mg/d)** |  |  |  |  |  |  |  |  |  |
| Sex Age |  |  |  |  |  |  |  |  |  |
| Men 19-45 y | 92.7% | 93.0% | 91.6% | 89.7% | 84.3% | 84.1% | 85.0% | <0.001 | <0.001 |
| Men 46+ y | 93.2% | 91.6% | 92.6% | 90.7% | 87.2% | 85.2% | 86.1% | <0.001 |  |
| Women 19-45 y | 96.6% | 96.5% | 95.0% | 96.0% | 94.1% | 92.0% | 90.2% | <0.001 |  |
| Women 46+ y | 95.6% | 97.4% | 93.5% | 95.0% | 92.7% | 90.9% | 92.2% | <0.001 |  |
| Socioeconomic status |  |  |  |  |  |  |  |  |  |
| High | 92.3% | 91.0% | 89.7% | 88.9% | 85.9% | 85.4% | 83.5% | <0.001 | <0.001 |
| Medium | 95.2% | 96.3% | 94.5% | 94.2% | 91.0% | 88.9% | 90.5% | <0.001 |  |
| Low | 97.8% | 98.0% | 97.1% | 98.1% | 95.0% | 93.5% | 96.1% | 0.001 |  |
| Race/ethnicity |  |  |  |  |  |  |  |  |  |
| Hispanic | 97.3% | 95.8% | 95.9% | 96.6% | 93.4% | 90.9% | 92.1% | <0.001 | <0.001 |
| Non-Hispanic white | 93.7% | 94.2% | 92.2% | 91.9% | 88.3% | 87.4% | 87.1% | <0.001 |  |
| Non-Hispanic black | 96.9% | 95.7% | 95.4% | 95.8% | 91.7% | 88.7% | 91.6% | <0.001 |  |
| Other race | 96.6% | 96.3% | 95.3% | 91.3% | 91.4% | 88.7% | 89.9% | <0.001 |  |
| BMI |  |  |  |  |  |  |  |  |  |
| Underweight | 90.6% | 94.5% | 94.3% | 94.9% | 87.1% | 91.0% | 88.9% | 0.401 | <0.001 |
| Normal weight | 94.7% | 94.0% | 92.1% | 92.7% | 87.5% | 85.8% | 86.0% | <0.001 |  |
| Overweight | 93.7% | 94.5% | 94.0% | 91.1% | 90.3% | 87.7% | 90.0% | <0.001 |  |
| Obese | 95.5% | 95.5% | 93.3% | 94.7% | 90.9% | 90.1% | 89.0% | <0.001 |  |
| **Vitamin K (µg/d)** |  |  |  |  |  |  |  |  |  |
| Sex Age |  |  |  |  |  |  |  |  |  |
| Men 19-45 y | 80.9% | 78.2% | 78.8% | 78.6% | 71.8% | 71.5% | 73.1% | <0.001 | <0.001 |
| Men 46+ y | 77.2% | 72.9% | 74.5% | 70.5% | 66.0% | 66.0% | 65.9% | <0.001 |  |
| Women 19-45 y | 72.3% | 68.9% | 71.3% | 70.3% | 62.8% | 57.6% | 57.7% | <0.001 |  |
| Women 46+ y | 68.1% | 60.1% | 63.8% | 59.7% | 51.4% | 52.2% | 53.3% | <0.001 |  |
| Socioeconomic status |  |  |  |  |  |  |  |  |  |
| High | 64.0% | 62.1% | 64.6% | 60.2% | 51.2% | 51.3% | 49.6% | <0.001 | <0.001 |
| Medium | 78.1% | 72.9% | 74.3% | 72.3% | 67.0% | 65.3% | 66.9% | <0.001 |  |
| Low | 87.4% | 83.6% | 82.7% | 84.4% | 77.8% | 76.5% | 80.9% | <0.001 |  |
| Race/ethnicity |  |  |  |  |  |  |  |  |  |
| Hispanic | 82.5% | 84.4% | 83.2% | 80.3% | 72.4% | 69.0% | 73.4% | <0.001 | <0.001 |
| Non-Hispanic white | 73.4% | 68.3% | 70.5% | 67.3% | 60.8% | 60.4% | 59.9% | <0.001 |  |
| Non-Hispanic black | 75.3% | 69.5% | 69.8% | 76.2% | 66.4% | 64.7% | 65.5% | <0.001 |  |
| Other race | 73.1% | 64.9% | 69.0% | 61.4% | 57.0% | 53.8% | 55.2% | <0.001 |  |
| BMI |  |  |  |  |  |  |  |  |  |
| Underweight | 63.0% | 63.6% | 73.2% | 67.0% | 71.5% | 47.5% | 55.2% | 0.137 | <0.001 |
| Normal weight | 73.8% | 68.6% | 67.2% | 67.1% | 60.3% | 56.4% | 57.0% | <0.001 |  |
| Overweight | 73.6% | 71.4% | 75.0% | 69.0% | 60.9% | 62.1% | 63.9% | <0.001 |  |
| Obese | 77.2% | 70.3% | 73.2% | 72.3% | 66.0% | 65.4% | 64.2% | <0.001 |  |
| **Choline (mg/d)** |  |  |  |  |  |  |  |  |  |
| Sex Age |  |  |  |  |  |  |  |  |  |
| Men 19-45 y | - | 80.8% | 83.4% | 80.6% | 82.0% | 82.1% | 83.9% | 0.266 | <0.001 |
| Men 46+ y | - | 85.1% | 85.6% | 83.9% | 84.4% | 85.8% | 83.6% | 0.570 |  |
| Women 19-45 y | - | 90.1% | 92.3% | 88.5% | 91.4% | 86.7% | 84.9% | <0.001 |  |
| Women 46+ y | - | 92.8% | 92.4% | 89.2% | 90.1% | 87.7% | 88.9% | 0.002 |  |
| Socioeconomic status |  |  |  |  |  |  |  |  |  |
| High | - | 86.4% | 88.6% | 85.1% | 86.8% | 84.9% | 84.7% | 0.121 | 0.447 |
| Medium | - | 87.7% | 88.8% | 85.7% | 87.0% | 86.2% | 85.8% | 0.035 |  |
| Low | - | 86.8% | 86.2% | 87.2% | 88.1% | 84.3% | 84.9% | 0.405 |  |
| Race/ethnicity |  |  |  |  |  |  |  |  |  |
| Hispanic | - | 85.8% | 86.1% | 82.8% | 83.9% | 83.8% | 81.8% | 0.030 | <0.001 |
| Non-Hispanic white | - | 87.1% | 88.6% | 85.6% | 87.4% | 86.5% | 86.0% | 0.220 |  |
| Non-Hispanic black | - | 89.0% | 89.6% | 87.3% | 87.5% | 83.0% | 87.7% | 0.047 |  |
| Other race | - | 87.6% | 91.6% | 88.9% | 88.1% | 85.3% | 85.0% | 0.050 |  |
| BMI |  |  |  |  |  |  |  |  |  |
| Underweight | - | 85.6% | 93.1% | 87.7% | 88.8% | 84.6% | 80.9% | 0.332 | 0.223 |
| Normal weight | - | 87.7% | 87.3% | 87.2% | 88.1% | 85.0% | 85.8% | 0.181 |  |
| Overweight | - | 86.9% | 90.7% | 85.0% | 85.6% | 86.3% | 85.7% | 0.067 |  |
| Obese | - | 87.2% | 87.2% | 84.9% | 87.4% | 85.6% | 85.3% | 0.217 |  |

Table S2 Intake of increased and decreased nutrients for US adults across seven surveys by age sex, socioeconomic status, race/ethnicity, and BMI in the National Health and Nutrition Examination Survey (NHANES 2003-2016)

|  | 2003-2004 | 2005-2006 | 2007-2008 | 2009-2010 | 2011-2012 | 2013-2014 | 2015-2016 | *P* trend | *P* value |
| --- | --- | --- | --- | --- | --- | --- | --- | --- | --- |
| **Alpha-carotene (µg/d)** |  |  |  |  |  |  |  |  |  |
| Sex Age |  |  |  |  |  |  |  |  |  |
| Men 19-45 y | 385.50 | 319.14 | 369.07 | 424.61 | 430.36 | 364.26 | 333.48 | 0.994 | <0.001 |
| Men 46+ y | 381.13 | 443.11 | 417.75 | 506.12 | 659.98 | 428.30 | 466.93 | 0.044 |  |
| Women 19-45 y | 362.92 | 329.04 | 354.87 | 374.24 | 411.44 | 377.83 | 419.03 | 0.117 |  |
| Women 46+ y | 407.18 | 444.88 | 470.15 | 450.72 | 485.48 | 497.99 | 412.69 | 0.512 |  |
| Socioeconomic status |  |  |  |  |  |  |  |  |  |
| High | 467.43 | 410.53 | 468.62 | 537.95 | 746.66 | 508.91 | 446.08 | 0.083 | <0.001 |
| Medium | 351.87 | 374.02 | 384.97 | 404.72 | 384.77 | 389.90 | 397.80 | 0.193 |  |
| Low | 331.10 | 316.39 | 298.27 | 328.45 | 303.17 | 269.89 | 330.25 | 0.661 |  |
| Race/ethnicity |  |  |  |  |  |  |  |  |  |
| Hispanic | 400.12 | 393.05 | 387.07 | 422.82 | 361.40 | 430.31 | 343.48 | 0.476 | <0.001 |
| Non-Hispanic white | 395.11 | 395.66 | 418.73 | 450.88 | 539.51 | 422.82 | 421.02 | 0.084 |  |
| Non-Hispanic black | 271.61 | 260.58 | 274.56 | 295.90 | 299.12 | 286.49 | 318.29 | 0.292 |  |
| Other race | 439.71 | 436.77 | 516.16 | 588.07 | 657.04 | 562.30 | 547.32 | 0.047 |  |
| BMI |  |  |  |  |  |  |  |  |  |
| Underweight | 157.77 | 403.97 | 325.43 | 498.38 | 384.51 | 359.96 | 450.81 | 0.166 | <0.001 |
| Normal weight | 419.17 | 402.36 | 455.41 | 438.21 | 621.42 | 493.83 | 407.31 | 0.085 |  |
| Overweight | 333.58 | 404.10 | 379.12 | 509.67 | 422.63 | 407.37 | 413.99 | 0.596 |  |
| Obese | 410.75 | 342.16 | 385.07 | 370.55 | 449.96 | 377.60 | 406.04 | 0.017 |  |
| **Beta-carotene (µg/d)** |  |  |  |  |  |  |  |  |  |
| Sex Age |  |  |  |  |  |  |  |  |  |
| Men 19-45 y | 1834.74 | 1649.95 | 1879.25 | 1844.56 | 2048.14 | 2008.54 | 1845.42 | 0.114 | <0.001 |
| Men 46+ y | 2192.32 | 2253.42 | 2193.98 | 2567.66 | 3134.94 | 2294.37 | 2359.12 | 0.086 |  |
| Women 19-45 y | 1881.30 | 1716.35 | 1759.01 | 1929.38 | 2155.54 | 2148.95 | 2220.88 | 0.001 |  |
| Women 46+ y | 2370.12 | 2508.85 | 2460.84 | 2615.77 | 2851.95 | 2649.89 | 2396.23 | 0.276 |  |
| Socioeconomic status |  |  |  |  |  |  |  |  |  |
| High | 2627.35 | 2382.81 | 2553.29 | 2851.06 | 3684.50 | 2694.91 | 2534.70 | 0.103 | <0.001 |
| Medium | 1858.41 | 1859.63 | 1920.04 | 2039.66 | 2084.72 | 2148.91 | 2115.97 | 0.001 |  |
| Low | 1587.15 | 1733.85 | 1446.40 | 1547.99 | 1450.44 | 1620.43 | 1560.83 | 0.739 |  |
| Race/ethnicity |  |  |  |  |  |  |  |  |  |
| Hispanic | 1729.27 | 1684.66 | 1718.83 | 1800.95 | 1746.67 | 2064.31 | 1730.42 | 0.157 | <0.001 |
| Non-Hispanic white | 2119.49 | 2063.75 | 2126.52 | 2328.66 | 2745.06 | 2269.34 | 2206.50 | 0.038 |  |
| Non-Hispanic black | 1920.05 | 1904.90 | 1930.85 | 1863.81 | 2110.08 | 2170.34 | 2268.18 | 0.007 |  |
| Other race | 2325.33 | 2462.56 | 2562.89 | 2882.26 | 3013.82 | 3026.39 | 3065.21 | 0.004 |  |
| BMI |  |  |  |  |  |  |  |  |  |
| Underweight | 1322.73 | 2420.22 | 1627.30 | 2416.59 | 2117.99 | 2191.03 | 2207.59 | <0.001 | <0.001 |
| Normal weight | 2183.81 | 2142.03 | 2299.77 | 2327.02 | 3094.56 | 2729.98 | 2420.11 | 0.051 |  |
| Overweight | 1864.62 | 2123.45 | 2063.28 | 2460.49 | 2459.71 | 2291.94 | 2212.37 | 0.166 |  |
| Obese | 2178.53 | 1803.05 | 1906.80 | 1968.08 | 2229.18 | 1958.30 | 2092.60 | 0.010 |  |
| **Beta-cryptoxanthin (µg/d)** |  |  |  |  |  |  |  |  |  |
| Sex Age |  |  |  |  |  |  |  |  |  |
| Men 19-45 y | 152.88 | 141.64 | 75.54 | 74.72 | 80.81 | 72.56 | 75.79 | <0.001 | <0.001 |
| Men 46+ y | 141.10 | 161.01 | 90.49 | 97.24 | 83.53 | 94.58 | 100.45 | <0.001 |  |
| Women 19-45 y | 127.16 | 108.48 | 61.02 | 75.38 | 73.17 | 74.68 | 73.56 | <0.001 |  |
| Women 46+ y | 145.18 | 124.65 | 82.51 | 100.30 | 85.97 | 92.76 | 93.46 | 0.001 |  |
| Socioeconomic status |  |  |  |  |  |  |  |  |  |
| High | 155.26 | 149.84 | 83.24 | 93.80 | 86.85 | 90.48 | 98.93 | <0.001 | <0.001 |
| Medium | 136.60 | 125.46 | 75.37 | 83.44 | 78.11 | 81.40 | 78.45 | <0.001 |  |
| Low | 131.50 | 123.15 | 69.03 | 90.29 | 79.83 | 76.19 | 103.22 | 0.015 |  |
| Race/ethnicity |  |  |  |  |  |  |  |  |  |
| Hispanic | 191.04 | 147.00 | 92.59 | 106.20 | 104.66 | 101.91 | 105.23 | 0.001 | <0.001 |
| Non-Hispanic white | 133.50 | 128.87 | 69.86 | 80.29 | 75.65 | 76.47 | 81.39 | <0.001 |  |
| Non-Hispanic black | 147.97 | 144.47 | 72.47 | 78.42 | 70.69 | 70.70 | 73.08 | <0.001 |  |
| Other race | 140.10 | 144.82 | 145.85 | 136.12 | 106.12 | 135.57 | 112.82 | 0.126 |  |
| BMI |  |  |  |  |  |  |  |  |  |
| Underweight | 194.24 | 178.09 | 55.19 | 93.36 | 63.60 | 72.22 | 69.46 | <0.001 | <0.001 |
| Normal weight | 149.19 | 143.86 | 87.43 | 89.28 | 83.71 | 98.53 | 94.20 | <0.001 |  |
| Overweight | 126.54 | 135.05 | 74.97 | 94.53 | 81.67 | 81.67 | 96.93 | <0.001 |  |
| Obese | 146.33 | 119.78 | 71.35 | 78.12 | 79.24 | 75.58 | 82.05 | <0.001 |  |
| **Lycopene (µg/d)** |  |  |  |  |  |  |  |  |  |
| Sex Age |  |  |  |  |  |  |  |  |  |
| Men 19-45 y | 8858.04 | 6834.22 | 7639.89 | 7037.01 | 6734.60 | 6127.34 | 6725.59 | <0.001 | <0.001 |
| Men 46+ y | 6819.10 | 5736.56 | 5919.28 | 5921.90 | 5497.07 | 5214.71 | 6104.36 | 0.123 |  |
| Women 19-45 y | 5766.77 | 4682.67 | 5185.52 | 4950.26 | 4708.80 | 4445.34 | 4231.88 | <0.001 |  |
| Women 46+ y | 4665.65 | 4602.21 | 4399.28 | 4305.27 | 4355.14 | 4104.04 | 4291.37 | 0.144 |  |
| Socioeconomic status |  |  |  |  |  |  |  |  |  |
| High | 7494.21 | 5791.26 | 6587.92 | 5742.28 | 5737.02 | 5541.78 | 6047.72 | 0.003 | <0.001 |
| Medium | 6207.48 | 5401.09 | 5475.65 | 5508.16 | 5164.20 | 4667.43 | 4999.95 | <0.001 |  |
| Low | 5520.72 | 4372.01 | 4893.23 | 4853.45 | 4509.87 | 4643.66 | 4306.02 | 0.214 |  |
| Race/ethnicity |  |  |  |  |  |  |  |  |  |
| Hispanic | 7273.55 | 5250.30 | 5606.91 | 5520.54 | 5372.15 | 5649.33 | 5295.56 | 0.014 | <0.001 |
| Non-Hispanic white | 6902.55 | 5831.91 | 6063.68 | 5726.94 | 5502.03 | 4951.92 | 5623.43 | <0.001 |  |
| Non-Hispanic black | 4240.39 | 3756.70 | 4697.32 | 4438.88 | 4607.62 | 4010.98 | 3962.06 | 0.729 |  |
| Other race | 4469.19 | 4491.00 | 4677.17 | 5314.89 | 4367.29 | 4949.48 | 4580.80 | 0.821 |  |
| BMI |  |  |  |  |  |  |  |  |  |
| Underweight | 4494.94 | 8462.41 | 5752.99 | 2938.46 | 7724.31 | 4102.70 | 5396.42 | 0.482 | <0.001 |
| Normal weight | 6616.70 | 5284.20 | 6175.43 | 5656.64 | 5263.25 | 5474.67 | 5796.34 | 0.130 |  |
| Overweight | 6352.91 | 5569.38 | 5620.87 | 5624.82 | 5620.16 | 4591.47 | 5172.19 | <0.001 |  |
| Obese | 6734.60 | 5373.30 | 5557.80 | 5469.63 | 4918.65 | 4898.71 | 5071.18 | 0.003 |  |
| **Lutein + zeaxanthin (µg/d)** |  |  |  |  |  |  |  |  |  |
| Sex Age |  |  |  |  |  |  |  |  |  |
| Men 19-45 y | 1283.46 | 1211.81 | 1339.26 | 1232.14 | 1439.35 | 1443.19 | 1482.06 | 0.006 | <0.001 |
| Men 46+ y | 1623.48 | 1582.08 | 1482.26 | 1680.84 | 2070.77 | 1626.63 | 1603.31 | 0.249 |  |
| Women 19-45 y | 1363.67 | 1232.85 | 1210.05 | 1368.53 | 1498.25 | 1645.78 | 1700.61 | 0.001 |  |
| Women 46+ y | 1580.46 | 1695.32 | 1599.21 | 1837.49 | 2084.00 | 1836.92 | 1729.96 | 0.022 |  |
| Socioeconomic status |  |  |  |  |  |  |  |  |  |
| High | 1915.12 | 1771.97 | 1771.34 | 1980.36 | 2529.40 | 1959.62 | 2031.66 | 0.043 | <0.001 |
| Medium | 1305.55 | 1256.34 | 1284.54 | 1393.79 | 1473.69 | 1523.87 | 1480.88 | 0.001 |  |
| Low | 922.12 | 1229.42 | 975.14 | 948.88 | 1023.03 | 1236.46 | 1021.86 | 0.447 |  |
| Race/ethnicity |  |  |  |  |  |  |  |  |  |
| Hispanic | 1087.62 | 1023.55 | 1034.27 | 1076.45 | 1233.19 | 1462.09 | 1260.38 | <0.001 | <0.001 |
| Non-Hispanic white | 1443.26 | 1428.52 | 1414.31 | 1584.51 | 1881.35 | 1608.85 | 1575.61 | 0.011 |  |
| Non-Hispanic black | 1754.44 | 1696.85 | 1563.44 | 1457.55 | 1823.84 | 1762.26 | 1895.30 | 0.187 |  |
| Other race | 1831.31 | 1658.52 | 1882.62 | 2039.60 | 1829.27 | 2126.46 | 2357.26 | 0.021 |  |
| BMI |  |  |  |  |  |  |  |  |  |
| Underweight | 1521.71 | 1254.12 | 1104.66 | 2197.87 | 1275.56 | 1844.95 | 1689.91 | 0.277 | <0.001 |
| Normal weight | 1444.03 | 1529.64 | 1517.46 | 1653.99 | 2172.98 | 1843.97 | 1825.43 | 0.018 |  |
| Overweight | 1341.51 | 1426.47 | 1405.65 | 1570.26 | 1775.95 | 1707.31 | 1692.87 | 0.004 |  |
| Obese | 1574.68 | 1340.01 | 1328.16 | 1367.61 | 1493.09 | 1433.57 | 1457.21 | 0.082 |  |
| **Caffeine (mg/d)** |  |  |  |  |  |  |  |  |  |
| Sex Age |  |  |  |  |  |  |  |  |  |
| Men 19-45 y | 181.43 | 185.73 | 170.94 | 165.12 | 143.89 | 157.96 | 137.97 | <0.001 | <0.001 |
| Men 46+ y | 233.94 | 218.43 | 228.49 | 218.55 | 218.98 | 195.63 | 236.02 | 0.444 |  |
| Women 19-45 y | 144.27 | 152.39 | 140.45 | 137.13 | 131.62 | 116.47 | 120.55 | <0.001 |  |
| Women 46+ y | 164.61 | 165.65 | 178.89 | 161.35 | 164.42 | 166.50 | 165.09 | 0.744 |  |
| Socioeconomic status |  |  |  |  |  |  |  |  |  |
| High | 193.24 | 187.45 | 204.55 | 186.32 | 172.53 | 170.42 | 188.70 | 0.069 | <0.001 |
| Medium | 176.82 | 181.93 | 168.73 | 167.25 | 164.92 | 153.89 | 158.92 | 0.002 |  |
| Low | 147.59 | 119.32 | 157.96 | 129.98 | 135.10 | 158.19 | 127.42 | 0.933 |  |
| Race/ethnicity |  |  |  |  |  |  |  |  |  |
| Hispanic | 112.27 | 112.35 | 107.21 | 106.25 | 111.16 | 115.47 | 113.58 | 0.629 | <0.001 |
| Non-Hispanic white | 204.82 | 206.55 | 210.79 | 201.89 | 193.72 | 186.50 | 199.59 | 0.036 |  |
| Non-Hispanic black | 79.76 | 77.53 | 80.10 | 77.73 | 74.93 | 74.29 | 70.47 | 0.041 |  |
| Other race | 173.56 | 166.86 | 142.91 | 119.78 | 133.92 | 124.58 | 129.13 | 0.002 |  |
| BMI |  |  |  |  |  |  |  |  |  |
| Underweight | 123.38 | 160.27 | 181.06 | 151.10 | 102.61 | 136.15 | 116.58 | 0.256 | <0.001 |
| Normal weight | 169.88 | 167.65 | 179.78 | 159.96 | 155.80 | 151.28 | 150.30 | 0.008 |  |
| Overweight | 178.13 | 195.23 | 183.45 | 185.46 | 167.56 | 162.70 | 169.80 | 0.003 |  |
| Obese | 192.77 | 177.28 | 173.42 | 167.60 | 173.58 | 163.90 | 177.20 | 0.527 |  |
| **Theobromine (mg/d)** |  |  |  |  |  |  |  |  |  |
| Sex Age |  |  |  |  |  |  |  |  |  |
| Men 19-45 y | 44.78 | 39.90 | 37.73 | 37.76 | 31.17 | 36.91 | 32.49 | 0.003 | <0.001 |
| Men 46+ y | 41.00 | 38.49 | 40.56 | 42.89 | 41.81 | 40.25 | 33.23 | 0.148 |  |
| Women 19-45 y | 45.71 | 38.79 | 39.93 | 34.41 | 36.01 | 35.17 | 29.59 | <0.001 |  |
| Women 46+ y | 35.37 | 33.78 | 37.10 | 38.28 | 34.77 | 37.51 | 33.91 | 0.976 |  |
| Socioeconomic status |  |  |  |  |  |  |  |  |  |
| High | 43.67 | 40.20 | 47.00 | 43.48 | 36.44 | 40.22 | 32.95 | <0.001 | <0.001 |
| Medium | 42.60 | 37.77 | 36.06 | 36.93 | 36.48 | 36.89 | 32.74 | 0.004 |  |
| Low | 26.69 | 24.39 | 27.87 | 29.46 | 29.23 | 30.05 | 26.52 | 0.469 |  |
| Race/ethnicity |  |  |  |  |  |  |  |  |  |
| Hispanic | 30.00 | 28.16 | 26.23 | 26.50 | 24.51 | 28.47 | 26.62 | 0.393 | <0.001 |
| Non-Hispanic white | 46.31 | 41.22 | 43.25 | 42.20 | 39.99 | 41.59 | 35.87 | <0.001 |  |
| Non-Hispanic black | 27.48 | 30.13 | 30.85 | 32.71 | 28.35 | 29.89 | 22.25 | 0.084 |  |
| Other race | 31.45 | 25.67 | 26.97 | 30.12 | 29.85 | 28.62 | 29.04 | 0.894 |  |
| BMI |  |  |  |  |  |  |  |  |  |
| Underweight | 21.61 | 35.43 | 44.31 | 45.13 | 46.83 | 46.83 | 33.17 | 0.102 | <0.001 |
| Normal weight | 43.34 | 38.55 | 43.77 | 40.24 | 35.97 | 39.36 | 32.15 | 0.001 |  |
| Overweight | 39.60 | 38.48 | 36.44 | 38.70 | 37.14 | 37.91 | 34.28 | 0.086 |  |
| Obese | 43.28 | 36.36 | 36.31 | 35.97 | 34.16 | 36.07 | 31.13 | 0.003 |  |

Table S3 Trends in dietary nutrient intake among US adults except participants with implausible energy in the National Health and Nutrition Examination Survey (NHANES 2003-2016)^1^

|  | 2003-2004 | 2005-2006 | 2007-2008 | 2009-2010 | 2011-2012 | 2013-2014 | 2015-2016 | *P* for linear trend^2^ | 2003-2016 | |  | | | | | | | | |
| --- | --- | --- | --- | --- | --- | --- | --- | --- | --- | --- | --- | --- | --- | --- | --- | --- | --- | --- | --- |
| Macro-nutrients |  |  |  |  |  |  |  |  |  | |  | | | | | | | | |
| Energy (kcal/d) | 2091.29±17.29 | 2068.81±19.43 | 2028.61±19.39 | 2031.80±12.40 | 2062.50±17.73 | 2013.19±11.26 | 2027.38±18.71 | 0.005 | 2045.42±6.28 | |  | | | | | | | | |
| Protein (g/d) | 80.75±1.11 | 81.39±0.66 | 79.80±0.84 | 80.28±0.74 | 80.08±0.68 | 81.00±0.64 | 80.40±0.87 | 0.755 | 80.52±0.30 | |  | | | | | | | | |
| Carbohydrate (g/d) | 253.94±2.26 | 249.38±2.65 | 246.23±2.39 | 247.48±1.38 | 251.24±2.11 | 238.36±1.62 | 235.49±2.31 | <0.001 | 245.80±0.81 | |  | | | | | | | | |
| Total fat (g/d) | 79.25±0.89 | 78.39±1.00 | 76.85±1.07 | 75.49±0.67 | 77.57±1.00 | 77.74±0.64 | 80.83±0.96 | 0.332 | 78.03±0.35 | |  | | | | | | | | |
| Total sugars (g/d) | 116.82±1.18 | 113.23±1.67 | 111.01±2.13 | 110.18±1.03 | 110.06±1.26 | 104.16±1.23 | 100.60±1.41 | <0.001 | 109.24±0.57 | |  | | | | | | | | |
| Dietary fiber (g/d) | 15.25±0.37 | 15.51±0.22 | 15.81±0.36 | 16.67±0.17 | 17.56±0.27 | 16.57±0.21 | 17.02±0.36 | <0.001 | 16.37±0.12 | |  | | | | | | | | |
| Saturated fatty acids (g/d) | 26.06±0.31 | 26.13±0.33 | 25.27±0.44 | 24.60±0.29 | 24.93±0.38 | 25.13±0.23 | 26.52±0.37 | 0.730 | 25.52±0.13 | |  | | | | | | | | |
| Monounsaturated fatty acids (g/d) | 29.70±0.36 | 28.77±0.35 | 28.36±0.38 | 27.11±0.25 | 27.73±0.38 | 27.10±0.23 | 28.37±0.36 | <0.001 | 28.14±0.13 | |  | | | | | | | | |
| Polyunsaturated fatty acids (g/d) | 16.79±0.22 | 16.71±0.29 | 16.53±0.21 | 16.88±0.15 | 18.56±0.24 | 18.12±0.19 | 18.58±0.24 | <0.001 | 17.48±0.08 | |  | | | | | | | | |
| Cholesterol (g/d) | 283.29±5.74 | 279.24±2.99 | 280.52±4.63 | 268.06±3.82 | 272.70±3.42 | 284.56±3.78 | 292.04±4.39 | 0.194 | 280.18±1.61 | |  | | | | | | | | |
| Minerals |  |  |  |  |  |  |  |  |  | |  | | | | | | | | |
| Calcium (mg/d) | 843.29±16.59 | 904.71±11.89 | 900.51±18.15 | 975.59±8.08 | 954.57±11.59 | 937.49±9.54 | 934.89±17.65 | <0.001 | 922.78±5.27 | |  | | | | | | | | |
| Phosphorus (mg/d) | 1283.15±18.81 | 1296.83±12.27 | 1290.59±17.04 | 1362.94±10.00 | 1362.89±13.50 | 1349.38±11.13 | 1350.44±18.65 | <0.001 | 1329.12±5.65 | |  | | | | | | | | |
| Magnesium (mg/d) | 270.24±4.94 | 290.35±3.01 | 285.46±5.07 | 296.00±2.36 | 300.60±4.10 | 293.43±3.32 | 298.49±4.67 | <0.001 | 290.98±1.59 | |  | | | | | | | | |
| Iron (mg/d) | 15.54±0.26 | 15.80±0.15 | 15.13±0.28 | 15.14±0.10 | 15.04±0.14 | 14.16±0.12 | 13.75±0.18 | <0.001 | 14.91±0.07 | |  | | | | | | | | |
| Zinc (mg/d) | 11.78±0.23 | 12.29±0.21 | 11.79±0.23 | 11.62±0.15 | 11.04±0.15 | 10.90±0.11 | 11.00±0.16 | <0.001 | 11.47±0.07 | |  | | | | | | | | |
| Copper (mg/d) | 1.22±0.02 | 1.35±0.01 | 1.30±0.02 | 1.28±0.01 | 1.27±0.03 | 1.19±0.01 | 1.23±0.02 | 0.004 | 1.26±0.01 | |  | | | | | | | | |
| Sodium (mg/d) | 3355.27±34.80 | 3366.60±33.28 | 3329.08±40.01 | 3466.71±22.55 | 3434.30±29.99 | 3388.85±21.47 | 3433.99±36.71 | 0.054 | 3397.56±11.96 | |  | | | | | | | | |
| Potassium (mg/d) | 2637.77±35.40 | 2637.44±30.11 | 2588.80±35.75 | 2679.42±18.73 | 2699.78±35.29 | 2578.71±29.66 | 2591.09±33.82 | 0.376 | 2630.00±11.94 | |  | | | | | | | | |
| Selenium (µg/d) | 107.23±1.55 | 107.77±1.06 | 107.68±1.29 | 109.90±0.99 | 111.40±0.95 | 113.79±0.87 | 112.85±1.18 | <0.001 | 110.18±0.44 | |  | | | | | | | | |
| Vitamins |  |  |  |  |  |  |  |  |  |  | | | | | | | | | |
| Vitamin A (µg/d) | 98.75±13.34 | 625.51±10.05 | 617.41±18.50 | 640.14±11.74 | 653.87±27.47 | 622.42±12.65 | 625.70±11.31 | 0.102 | 626.60±6.10 | |  | | | | | | | | |
| Vitamin B_1_ (mg/d) | 1.62±0.02 | 1.64±0.01 | 1.60±0.03 | 1.63±0.01 | 1.58±0.01 | 1.57±0.01 | 1.55±0.02 | <0.001 | 1.60±0.01 | |  | | | | | | | | |
| Vitamin B_2_(mg/d) | 2.19±0.04 | 2.20±0.02 | 2.14±0.04 | 2.10±0.02 | 2.08±0.03 | 2.11±0.03 | 2.12±0.03 | 0.024 | 2.13±0.01 | |  | | | | | | | | |
| Vitamin B_3_ (mg/d) | 23.79±0.37 | 25.00±0.20 | 24.67±0.34 | 25.36±0.13 | 25.08±0.27 | 25.69±0.22 | 25.34±0.32 | <0.001 | 25.01±0.11 | |  | | | | | | | | |
| Vitamin B_6_ (mg/d) | 1.84±0.03 | 1.99±0.02 | 1.98±0.03 | 2.08±0.01 | 2.11±0.04 | 2.17±0.04 | 2.08±0.03 | <0.001 | 2.01±0.01 | |  | | | | | | | | |
| Vitamin B_12_ (µg/d) | 5.14±0.18 | 5.52±0.13 | 5.32±0.14 | 5.27±0.07 | 5.06±0.14 | 4.86±0.08 | 4.95±0.14 | 0.006 | 5.16±0.05 | |  | | | | | | | | |
| Folate DFE (µg/d) | 527.74±10.19 | 535.89±6.81 | 533.36±13.20 | 538.62±4.58 | 544.83±5.91 | 509.32±5.55 | 505.73±9.95 | 0.021 | 527.62±3.17 | |  | | | | | | | | |
| Vitamin C (mg/d) | 85.66±2.74 | 84.93±1.85 | 83.24±3.23 | 83.85±1.44 | 83.12±3.26 | 77.90±1.46 | 77.25±2.18 | 0.002 | 82.17±0.89 | |  | | | | | | | | |
| Vitamin D (µg/d) | NA | NA | 4.29±0.10 | 5.01±0.10 | 4.54±0.11 | 4.65±0.11 | 4.51±0.10 | 0.885 | 4.60±0.04 | |  | | | | | | | | |
| Vitamin E (mg/d) | 6.80±0.12 | 7.13±0.11 | 7.36±0.17 | 7.72±0.09 | 8.58±0.15 | 8.92±0.14 | 8.92±0.22 | <0.001 | 7.95±0.06 | |  | | | | | | | | |
| Vitamin K (µg/d) | 93.21±2.26 | 101.69±3.44 | 96.52±3.86 | 101.92±2.21 | 131.49±11.98 | 17.10±2.52 | 118.48±3.80 | 0.102 | 109.03±2.11 | |  | | | | | | | | |
| Choline (mg/d) | NA | 320.81±2.28 | 314.80±4.03 | 327.12±3.72 | 321.74±3.61 | 325.77±4.02 | 329.01±3.34 | 0.012 | 323.32±1.46 | |  | | | | | | | | |
| Alpha-carotene (µg/d) | 377.70±17.50 | 384.25±16.94 | 403.02±27.48 | 432.62±19.51 | 499.83±50.63 | 415.10±19.48 | 406.05±27.70 | 0.042 | 417.60±10.80 | |  | | | | | | | | |
| Beta-carotene (µg/d) | 2040.07±74.87 | 2038.14±65.96 | 2063.46±108.40 | 2218.03±61.83 | 2570.93±186.98 | 2244.35±85.35 | 2201.19±80.62 | 0.012 | 2200.33±40.04 | |  | | | | | | | | |
| Beta-cryptoxanthin (µg/d) | 138.91±7.09 | 129.53±5.06 | 76.56±5.22 | 85.59±4.84 | 80.41±2.28 | 83.30±2.71 | 86.19±4.73 | <0.001 | 96.48±1.79 | |  | | | | | | | | |
| Lycopene (µg/d) | 6263.32±377.45 | 5225.78±153.77 | 5517.99±140.69 | 5387.58±205.04 | 5166.07±187.62 | 4772.70±111.24 | 5123.06±153.62 | <0.001 | 5337.03±73.44 | |  | | | | | | | | |
| Lutein + zeaxanthin (µg/d) | 1440.69±60.54 | 1428.32±53.44 | 1384.99±70.10 | 1506.77±52.03 | 1786.48±111.61 | 1611.18±51.30 | 1623.87±68.26 | 0.002 | 1543.77±27.12 | |  | | | | | | | | |
| Caffeine (mg/d) | 177.14±6.98 | 177.67±5.64 | 175.50±8.92 | 166.98±6.39 | 165.10±7.10 | 157.42±4.89 | 165.91±4.25 | 0.007 | 169.16±2.45 | |  | | | | | | | | |
| Theobromine (mg/d) | 39.98±1.35 | 36.64±1.51 | 36.75±1.41 | 35.78±1.02 | 35.27±1.63 | 35.62±1.22 | 31.77±1.13 | <0.001 | 35.88±0.52 | |  | | | | | | | | |
| Percentage of energy |  |  |  |  |  |  |  |  |  | |  | | | | | | | | |
| Protein (%,kcal) | 16.6% | 17.1% | 17.4% | 17.3% | 16.9% | 17.7% | 17.5% | 0.004 | 17.2% | |  | | | | | | | | |
| Carbohydrate (%,kcal) | 52.8% | 53.2% | 54.4% | 54.0% | 53.4% | 53.0% | 51.8% | 0.170 | 53.2% | |  | | | | | | | | |
| Total fat (%,kcal) | 36.4% | 36.8% | 37.5% | 36.4% | 36.3% | 38.2% | 39.3% | <0.001 | 37.3% | |  |  |  |  |  |  |  |  |  |
| Total Sugars (%,kcal) | 24.3% | 24.2% | 24.6% | 24.1% | 23.6% | 23.3% | 22.4% | <0.001 | 23.8% | |  | | | | | | | | |
| Saturated fatty acids (%,kcal) | 12.6% | 12.9% | 13.0% | 12.5% | 12.3% | 13.0% | 13.5% | 0.007 | 12.8% | |  | | | | | | | | |
| Monounsaturated fatty acids (%,kcal) | 13.9% | 13.7% | 14.1% | 13.4% | 13.2% | 13.6% | 14.1% | 0.841 | 13.7% | |  | | | | | | | | |
| Polyunsaturated fatty acids (%,kcal) | 8.2% | 8.4% | 8.6% | 8.6% | 9.1% | 9.3% | 9.5% | <0.001 | 8.8% | |  | | | | | | | | |

^1^Values are survey-weighted means ± SEs, determined with adjustment for the complex sampling design of NHANES.

Except participants in the range of implausible energy intake: <500 or >3500 Kcal/d for women and <800 or >4000 Kcal/d for men.

^2^P trend for the overall F test for each variable from survey-weighted linear regression.

Table S4 Trends in percentage of dietary nutrient intake not meeting Dietary Reference Intakes among US adults except participants with implausible energy in the National Health and Nutrition Examination Survey (NHANES 2003-2016)^1^

|  | 2003-2004 | 2005-2006 | 2007-2008 | 2009-2010 | 2011-2012 | 2013-2014 | 2015-2016 | *P* for linear trend^2^ | 2003-2016 |
| --- | --- | --- | --- | --- | --- | --- | --- | --- | --- |
| Macro-nutrients |  |  |  |  |  |  |  |  |  |
| Energy (kcal/d)***** | 38.0% | 35.7% | 25.1% | 27.7% | 29.0% | 27.5% | 26.5% | <0.001 | 29.8% |
| Protein (kcal/d)*** | 21.0% | 19.8% | 18.5% | 17.7% | 20.7% | 19.2% | 19.6% | 0.496 | 19.5% |
| Carbohydrate (kcal/d)*** | 67.4% | 67.7% | 65.7% | 64.1% | 65.1% | 65.3% | 65.8% | 0.022 | 65.9% |
| Total Fat (kcal/d)*** | 60.9% | 60.9% | 62.2% | 58.8% | 56.4% | 61.0% | 61.3% | 0.461 | 60.2% |
| Total Sugars (kcal/d)**** | 88.2% | 87.5% | 88.7% | 88.2% | 86.5% | 87.0% | 85.0% | 0.004 | 87.3% |
| Dietary fiber (g/d)* | 92.9% | 92.4% | 91.2% | 88.8% | 86.3% | 89.4% | 88.1% | <0.001 | 89.8% |
| Saturated fatty acids(kcal/d)**** | 62.2% | 65.1% | 65.8% | 63.5% | 60.9% | 67.2% | 69.5% | 0.001 | 65.0% |
| Minerals |  |  |  |  |  |  |  |  |  |
| Calcium (mg/d) | 72.7% | 68.2% | 69.5% | 63.0% | 64.1% | 66.0% | 65.6% | <0.001 | 66.9% |
| Iron (mg/d) | 32.0% | 30.1% | 31.2% | 31.6% | 30.3% | 33.3% | 34.8% | 0.014 | 31.9% |
| Magnesium (mg/d) | 83.6% | 78.3% | 79.0% | 76.8% | 74.3% | 77.0% | 75.8% | <0.001 | 77.8% |
| Phosphorus(mg/d) | 9.6% | 9.3% | 8.9% | 6.5% | 7.5% | 7.2% | 8.0% | 0.008 | 8.1% |
| Potassium (mg/d)* | 96.3% | 96.4% | 97.1% | 96.6% | 96.4% | 96.9% | 97.3% | 0.070 | 96.7% |
| Sodium (mg/d)** | 76.5% | 76.9% | 76.3% | 80.8% | 80.4% | 80.3% | 79.0% | 0.001 | 78.6% |
| Zinc (mg/d) | 36.8% | 37.1% | 39.1% | 38.0% | 41.1% | 42.5% | 42.0% | 0.001 | 39.6% |
| Copper (mg/d) | 29.6% | 23.0% | 25.6% | 25.6% | 29.1% | 31.6% | 30.9% | 0.001 | 28.0% |
| Selenium (µg/d) | 9.7% | 9.8% | 10.3% | 8.1% | 8.3% | 7.9% | 7.7% | <0.001 | 8.8% |
| Vitamins |  |  |  |  |  |  |  |  |  |
| Vitamin A (µg/d) | 77.2% | 74.2% | 73.6% | 74.0% | 73.6% | 73.8% | 75.% | 0.420 | 74.6% |
| Vitamin B_1_ (mg/d) | 26.9% | 27.0% | 27.8% | 25.2% | 27.7% | 28.3% | 29.7% | 0.041 | 27.6% |
| Vitamin B_2_(mg/d) | 12.1% | 12.6% | 13.2% | 14.4% | 14.8% | 13.6% | 14.1% | 0.048 | 13.6% |
| Vitamin B_3_ (mg/d) | 17.5% | 15.7% | 16.8% | 13.9% | 14.6% | 14.0% | 16.2% | 0.079 | 15.5% |
| Vitamin B_6_(mg/d) | 35.1% | 31.3% | 31.4% | 28.8% | 28.4% | 27.8% | 31.0% | 0.003 | 30.5% |
| Vitamin B_12_ (µg/d) | 21.0% | 19.9% | 19.2% | 17.8% | 20.5% | 22.8% | 24.0% | 0.001 | 20.8% |
| Folate DFE (µg/d) | 38.1% | 37.5% | 38.1% | 36.1% | 36.4% | 40.8% | 42.6% | 0.008 | 38.6% |
| Vitamin C (mg/d) | 59.4% | 59.9% | 60.6% | 59.5% | 61.7% | 62.8% | 64.4% | 0.002 | 61.3% |
| Vitamin D (µg/d) | NA | NA | 97.9% | 96.7% | 97.4% | 96.3% | 97.1% | 0.086 | 97.1% |
| Vitamin E (mg/d) | 95.7% | 95.5% | 94.3% | 94.3% | 90.7% | 89.6% | 89.6% | <0.001 | 92.7% |
| Vitamin K (µg/d)* | 75.4% | 70.7% | 72.8% | 70.6% | 63.4% | 61.9% | 62.6% | <0.001 | 68.0% |
| Choline (mg/d) | NA | 89.2% | 90.2% | 87.7% | 89.0% | 87.5% | 86.7% | 0.001 | 88.4% |

^1^Except participants in the range of implausible energy intake: <500/>3500 Kcal/day for women and <800/>4000 Kcal/day for men.

^2^P trend for the overall F test for each variable from survey-weighted linear regression.

*Not meeting Adequate Intakes (AI).

**Exceeding Upper Intake Level (UL).

***Not meeting Acceptable Macronutrient Distribution Range (AMDR).

****Not meeting 2015-2020 Dietary Guidelines recommended limit (DGA). Values are means, determined with adjustment for the complex sampling design of NHANES.

Figure S1 Flowchart

Figure S2 Trends for dietary alpha-carotene (A1–A4), beta-carotene (B1–B4), lycopene (C1–C4), and caffeine (D1–D4) intake by age sex, socioeconomic status, race/ethnicity, and body mass index (BMI) for other nutrients intake in US adults across seven surveys in the National Health and Nutrition Examination Survey (NHANES 2003-2016).
